# Multi-omics analysis reveals prognostic value of tumor mutation burden in hepatocellular carcinoma

**DOI:** 10.1186/s12935-021-02049-w

**Published:** 2021-07-03

**Authors:** Qianhui Xu, Hao Xu, Rongshan Deng, Zijie Wang, Nanjun Li, Zhixuan Qi, Jiaxin Zhao, Wen Huang

**Affiliations:** 1 The Second Affiliated Hospital and Yuying Children’s Hospital of Wenzhou Medical University, No 109. Xueyuan West Road, Wenzhou, 325000 Zhejiang China; 2 Zhejiang University School of Medicine, Hangzhou, 310009 Zhejiang China

**Keywords:** Tumor mutation burden, Hepatocellular carcinoma, Tumor immune microenvironment, Immunotherapy

## Abstract

**Background:**

Hepatocellular carcinoma (HCC) was the sixth common malignancies characteristic with highly aggressive in the world. It was well established that tumor mutation burden (TMB) act as indicator of immunotherapeutic responsiveness in various tumors. However, the role of TMB in tumor immune microenvironment (TIME) is still obscure.

**Method:**

The mutation data was analyzed by employing “maftools” package. Weighted gene co-expression network analysis (WGCNA) was implemented to determine candidate module and significant genes correlated with TMB value. Differential analysis was performed between different level of TMB subgroups employing R package “limma”. Gene ontology (GO) enrichment analysis was implemented with “clusterProfiler”, “enrichplot” and “ggplot2” packages. Then risk score signature was developed by systematical bioinformatics analyses. K-M survival curves and receiver operating characteristic (ROC) plot were further analyzed for prognostic validity. To depict comprehensive context of TIME, XCELL, TIMER, QUANTISEQ, MCPcounter, EPIC, CIBERSORT, and CIBERSORT-ABS algorithm were employed. Additionally, the potential role of risk score on immune checkpoint blockade (ICB) immunotherapy was further explored. The quantitative real-time polymerase chain reaction was performed to detect expression of HTRA3.

**Results:**

TMB value was positively correlated with older age, male gender and early T status. A total of 75 intersection genes between TMB-related genes and differentially expressed genes (DEGs) were screened and enriched in extracellular matrix-relevant pathways. Risk score based on three hub genes significantly affected overall survival (OS) time, infiltration of immune cells, and ICB-related hub targets. The prognostic performance of risks score was validated in the external testing group. Risk-clinical nomogram was constructed for clinical application. HTRA3 was demonstrated to be a prognostic factor in HCC in further exploration. Finally, mutation of TP53 was correlated with risk score and do not interfere with risk score-based prognostic prediction.

**Conclusion:**

Collectively, a comprehensive analysis of TMB might provide novel insights into mutation-driven mechanism of tumorigenesis further contribute to tailored immunotherapy and prognosis prediction of HCC.

**Supplementary Information:**

The online version contains supplementary material available at 10.1186/s12935-021-02049-w.

## Introduction

Primary liver cancer is one of the most prevalent and aggressive malignancies whose incidence has raised rapidly in the world [1–3]. According to histopathological classification, approximately 80% of liver cancer cases were hepatocellular carcinoma (HCC) [2]. Such underlying risk factors for HCC as infections of hepatitis virus, aflatoxin exposure, type 2 diabetes, heavy alcohol intake and obesity played key role in leading to hepatocarcinogenesis [3]. Since sophisticated molecular-level diversity including genomics and genetic variation, HCC was considered as malignant disease with high heterogeneity from not only intertumoral but also intratumor standpoint [4–7]. Given that its high heterogeneity and etiologies differs well among different patients, tumor‐node‐metastasis (TNM) staging as widely used clinical classification achieved little in predicting overall survival and clinical outcome [8–10]. It is imperative, therefore, to generate robust tools for prognosis prediction and therapeutic response assessment, further facilitate precision and individualized treatment.

Recently, advances in such immunotherapy as anti-CTLA-4 and PD-1/L1 (immune checkpoint blockade, ICB) have exerted encouraging therapeutic efficacy subsequently elevated overall survival (OS) probability in multiple human malignancies [11–14]. Clinical trial data reported that 31% patients were obtained objective response to ICB treatment, which ignite people interest in immunological treatment against HCC [15, 16]. Traditionally, the tumor progression has been considered as a multistep process that only involves the genetic and epigenetic variation in tumor cells. With the deepening of research, it’s well established that signaling and secretions mediated by multiple cell subpopulations from tumor immune microenvironment (TIME) serves a key player in driving tumor progression, tolerance and evasion [17, 18].

Tumor mutation burden (TMB), representing the somatic coding errors such as base substitutions, deletions across, or insertions per million bases, has been termed as a promising indicator for predicting responsiveness to ICB based on numerous researches [19–21]. High TMB was found to promote antigens formation and subsequent infiltration of immune cells then strengthen immune response, which can lead to improved immunotherapeutic efficacy [22]. To date, there have been multiple studies focusing the correlation of TMB and immunotherapy in diverse cancers, including HCC [19, 20, 23, 24]. However, it is little to know the underlying functions of TMB related molecules in prognosis and immunotherapeutic efficacy of HCC. Thus, the most efficient method for accurate predictions of how a given tumor will respond to treatment or progress may be one based on molecular risk classification, recognizing HCC samples on line with specific molecular signatures, enhancing prognostic predictive precision and facilitate clinical-decision making accordingly.

Herein, this research was designed to elucidate the potential significance of TMB related molecules in HCC. Expression profiling matrix, clinical information and corresponding copy number variation data were obtained from TCGA portal. Firstly, landscape of somatic mutation was delineated using R package “maftools”. Then, differentially expressed genes (DEGs) were applied into identification TMB candidate genes based on WGCNA analysis of TMB-related genes. Next, candidate genes were further screened using LASSO regression analysis and final 3 hub genes were determined. Besides, multi-genes prognostic signature and risk-clinical nomogram was constructed then validated. Additionally, the potential role of risk signature in TIME and immunotherapy was explored. Moreover, the potential role of HTRA3 was explored in HCC. Finally, the synergistic effect of risk score with gene mutation was demonstrated. These findings may contribute novel insight into potential targets and advance precision immunotherapy for HCC.

## Materials and methods

### Collection of muti-omics information

Four categories of somatic mutation data of HCC samples were obtained from The Cancer Genome Atlas (TCGA) portal. The mutation files obtained through the “varscan variant aggregation and masking” platform was singled out for subsequent analysis. The Mutation Annotation Format (MAF) of somatic variants was prepared within the “maftools” [25] R package. Furthermore, gene expression profiling for HCC sample compared with normal tissues were obtained from TCGA-LIHC project. The corresponding clinical data were also obtained from the TCGA portal as descripted previously. The corresponding expression profiling information and the clinical data were downloaded from the ICGC (https://dcc.icgc.org). The detailed clinical data of HCC patients from TCGA-LIHC and ICGC-LIRI-JP were recorded in Additional file 1: Table S1. There was no necessity to obtain Ethics Committee approval since all information were publicly available and open-access. The analysis process flow chart was presented in Additional file 2: Figure S1.

### Detection of TMB and prognostic analysis

In this study, a Perl script was employed to fetch the somatic mutation data then the TMB scores for each sample was calculated through dividing the number of somatic mutations by the total length of exons (38 million). Additional file 1: Table S2 recorded the details of estimated TMB value of HCC patients. Subsequently, the median value was employed as the cutoff value to category HCC samples into high- and low-TMB subgroups.

Next, the calculated TMB information was integrated with corresponding follow-up information. The log-rank test was analyzed to determine prognostic difference between low- and high-TMB subgroups. Besides, the correlation of TMB values with clinicopathological variables was explored, Wilcoxon rank-sum test was analyzed between two groups of clinical characteristics, whereas Kruskal–Wallis (K-W) test was utilized among three or more groups.

### Weighted gene co-expression network analysis

The gene-expression profiles of total 56,753 genes were applied to explore the TMB-related modules using R package “WGCNA”. The correlations between sample traits and candidate modules are computed to determine the models highly correlated with traits, in which the genes are further analyzed to screen hub genes [26]. TMB value was employed as sample phenotype and a suited value of β was applied to build a scale‐free network. Then, a weighted adjacency matrix was converted to a topological overlap matrix (TOM) that measures the network connectivity of a gene. Genes with similar expression profiles were classified into different modules using hierarchical agglomerative clustering analysis, and the cutHeight value was set to 0.8. Module eigengenes (MEs) identifies expression patterns of all genes as a single characteristic expression profile within a given module. Besides, correlation analysis between module characteristic genes and sample traits was implemented by Pearson’s correlation test (*p < 0.05). Lastly, modules with the highest correlation were selected for further analyses.

### Identification of DEGs

Taking advantage of the “Limma” package with|log2FC|> 1 and False Discovery Rate (FDR) < 0.05, the differentially expressed genes (DEGs) between low- and high- TMB groups were screened. With the help of package “pheatmap”, heatmap was plotted to present the expression difference.

### Functional annotation

The intersection of genes in highest significant module with DEGs were introduced into further study. By using R package “org.Hs.eg.db”, the Entrez ID for each gene was obtained and the Gene ontology (GO) and Kyoto Encyclopedia of Genes and Genomes (KEGG) pathways analysis was performed with “clusterProfiler”, “enrichplot” and “ggplot2” packages and visualized the results.

### Construction of multi-genes prognostic signature

HCC patients with missing OS values or OS = 0 day were excluded in order to reduce statistical bias in our analysis. Finally, a TCGA cohort involving 365 patients was employed as training group. By using package “glment”, LASSO regression algorithm with package “glment” was analyzed. Then, three hub genes were determined and introduced into a prognostic risk signature. The risk score of each sample was obtained as the following equation: risk score = sum of risk coefficients * expression level of gene.

### Validation of the multi-genes prognostic signature

First, K–M survival analyses were performed with “survival” R package. Furthermore, univariate and multivariate Cox regression were employed for prognostic validity of risk score as an independent indicator. Subsequently, the receiver operating characteristic (ROC) curves were plotted to estimate the prognostic value. The ICGC-LIRI-JP dataset from the ICGC database was used as an independent validation cohort (n = 231). The prognostic predictive precision was further validated in the external validation group.

### Risk score with clinical features

To elucidate the clinical significance of risk score, the correlation analysis between risk score with such main clinicopathological variables as gender, age, pathological staging, and TNM categories was performed. To further validate whether Multi-genes prognostic signature remained great prognostic validity when HCC samples assigned into distinct subgroups according to clinical characteristics, stratification survival analysis were conducted.

### Risk score with TIME characterization

To uncover the correlation between the risk score and tumor infiltrating immune cells, the seven methods including XCELL, TIMER, QUANTISEQ, MCPcounter, EPIC, CIBERSORT, and CIBERSORT-ABS were used to evaluate the immune infiltrating situation. Spearman correlation was analyzed to explore the relevance between risk score and the immune infiltration statues. The differences in immune infiltrating cell fraction were compared between low and high-risk subgroups.

### Role of risk score in immune checkpoint blockade treatment

According to previous research, expression patterns of immune checkpoint blockade-related hub targets might contribute into efficacy of immunotherapy administration [27]. An increasing number of recognized immune checkpoints act to coordinately influence the local tumor-immune environment. In this study, six hub genes of immunotherapy: programmed death ligand 1 (PD‐L1, also known as CD274), programmed death 1 (PD‐1, also known as PDCD1), programmed death ligand 2 (PD‐L2, also known as PDCD1LG2), cytotoxic T‐lymphocyte antigen 4 (CTLA‐4), T‐cell immunoglobulin domain and mucin domain‐containing molecule‐3 (TIM‐3, also known as HAVCR2), and indoleamine 2,3‐dioxygenase 1 (IDO1) were fetched in HCC [28–31]. To further explore the potential role of risk signature in immunotherapy, correlation of prognostic signature with expression value of six ICB hub genes was analyzed. To reveal the potential role of risk score in response to immunotherapy, the expression values of 47 ICB-related hub targets (i.e., PDCD1, etc.,) were detected for further analysis.

### Development of prognostic nomogram

To comprehensively estimate prognostic ability of risk score, clinical stage, gender, age and tumor grade for 1‐, 2-, 3‐, 4‐, 5‐and 6‐year overall survival, time-dependent receiver operating characteristic (ROC) curves was performed to compute the area under the curve (AUC) values [32]. To construct a quantitative risk model to predicting overall survival rate, a nomogram including risk score and other clinical variables to predict 1/2/3-OS probability. Subsequently, the calibration curve which shown the prognostic value of as-constructed nomogram was developed.

### Cell culture

The human normal hepatocyte cell line SQG-7701 and four HCC cell lines MHCC-97H, Hep-3B, HCC-LM3, HepG2 purchased from the Cell Bank of the Type Culture Collection of the Chinese Academy of Sciences, Shanghai Institute of Biochemistry and Cell Biology. The cells were all cultured in Dulbecco’s Modified Eagle’s Medium (DMEM, Gibco BRL, Grand Island, NY, USA) supplemented with 10% fetal bovine serum (FBS; Invitrogen, Carlsbad, CA, USA) and antibiotics (100 μg/mL streptomycin and 100 U/mL penicillin, Sigma, St-Louis, MO, USA) in a humidified incubator containing 5% CO2 at 37 °C.

### Quantitative real-time polymerase chain reaction (qRT-PCR)

Total RNA was extracted from cells using TRIzol (Invitrogen, Carlsbad, CA, USA) according to provided instructions. RNA concentration and purity were measured in triplicates utilizing the NanoDrop 2000 spectrophotometer (Thermo Scientific Inc., Waltham, MA, 93 USA). Then, total RNA was reverse transcribed to cDNA using the cDNA Reverse Transcription Kit (Vazyme, Nanjing, China). qRT-PCR analyses were performed using SYBR® Premix Ex Taq™ II (Takara, Dalian, China) and Taqman UniversalMaster Mix II (Life Technologies Corporation, Carlsbad, CA, United States) on Applied Biosystems 7500/7500 Fast Real-Time PCR System (Thermo Fisher Scientific). The 2-ΔΔCt method was used to calculate the relative mRNA expression normalized by GAPDH and HTRA3. The sequences of primers used for PCR were as follows: HTRA3, 5′- AAGAAGTCGGACATTGCCACCATC -3′ (forward) and 5′- CCGTTGTCACTGTGTTCTGTAGGG -3′ (reverse); and GAPDH, 5′-GGAGCGAGATCCCTCCAAAAT-3′ (forward) and 5′- GGCTGTTGTCATACTTCTCATGG-3′ (reverse).

### Statistical analysis

Wilcoxon rank-sum test was a non-parametric statistical hypothesis test mainly used for comparisons between two groups and Kruskal–Wallis test was suitable for two or more categories. Overall survival (OS) refers to the interval from the date of diagnosis to the date of death. Survival curves were plotted via the Kaplan–Meier log rank test. CIBERSORT algorithm results with p ≥ 0.05 were rejected for further analysis. Univariate and multivariate analyses were performed via Cox regression models to validate the independent prognosis predictive performance of risk signature. The prognostic value for 1-, 2- and 3-year OS was assessed with the ROC curves. p < 0.05 deemed statistical significance. R software (version 4.0.2) was utilized for all statistical analyses.

## Results

### Landscape of somatic mutations in HCC

**Fig. 1:**
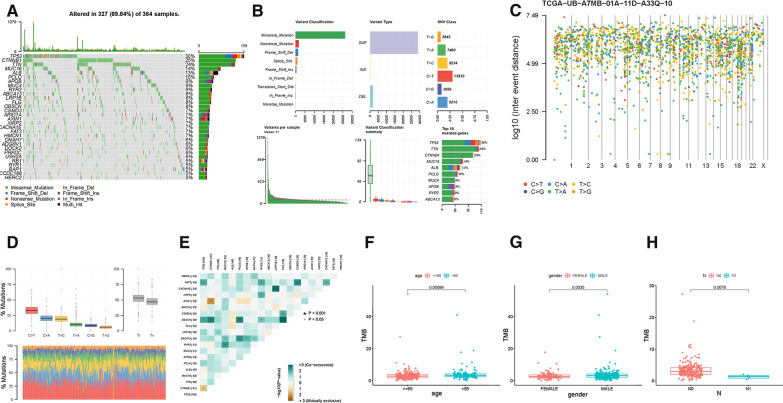
Landscape of somatic mutation profiles in HCC samples. **A** Mutation information of each gene in each sample was shown in the waterfall plot, where different colors with specific annotations at the bottom meant the various mutation types. The barplot above the legend exhibited the number of mutation burden. **B** Cohort summary plot displaying distribution of variants according to variant classification, type and SNV class. Bottom part (from left to right) indicates mutation load for each sample, variant classification type. A stacked barplot shows top ten mutated genes. **C** Rainfall plot of TCGA HCC sample TCGA − UB − A7MB − 01A − 11D − A33Q − 10. Each point is a mutation color coded according to SNV class. **D** Transition and transversion plot displaying distribution of SNVs in HCC classified into six transition and transversion events. Stacked bar plot (bottom) shows distribution of mutation spectra for every sample in the MAF file. **E** The coincident and exclusive associations across mutated genes. The correlation of TMB with age (**F**), gender (**G**) and T status (**H**)

As summarized in the waterfall map, 327 out of 364 HCC patients had the somatic mutation altered, accounting for 89.84%. And the results showed that TP53, CTNNB1 and TTN mutations are the highest three mutated genes in HCC samples, frequency was 30%, 25% and 24%, respectively (Fig. 1A). Missense mutations occupied an absolute position among the total mutation classification (Fig. 1Ba), single nucleotide polymorphism (SNP) accounted for more proportion than deletion (DEL) or insertion (INS, Fig. 1Bb and e). Meanwhile, C > T had the highest frequency, 13,933 times, in variant types of SNV (Fig. 1Bc, 1D). Figure 1Bd presented that the number of variants per sample and the median value of mutations variants was 71. Besides, the top 10 genetical variated genes were TP53, TTN, CTNNB1, MUC16, ALB, PCLO, MUC4, APOB, RYR2 and ABCA13 (Fig. 1Bf). Cancer genomes are characterized by genomic loci with localized hyper-mutations. Such hyper mutated genomic regions can be visualized by plotting inter variant distance on a linear genomic scale. These plots generally called rainfall plots. The rainfall plot of sample TCGA − UB − A7MB − 01A − 11D − A33Q − 10 was presented in Fig. 1C. Each dot represented SNV mutation type with corresponding color. To further elucidate the intrinsic connection between these genetic altered genes, the exclusive and co-occurrence correlation were presented in Fig. 1E. CACNA1E and LRP1B experienced highest co-occurrence frequency, while AXIN1 and CTNNB1 showed obvious mutuality of mutually exclusive.

### Clinical role of TMB in prognosis

When setting the cutoff value as median TMB value, HCC samples were assigned into two groups, namely, TMB low group with 180 patients and TMB high group with 182 patients (Additional file 1: Table S2). Besides, K-M survival analysis was plotted to identify the prognostic difference of TMB. Likewise, previous research pointed out that higher level of TMB facilitate tumor elimination further leads to longer survival [19, 21, 33]. There was no statistical difference of log-rank test (Additional file 2: Figure S2A, P = 0.108), however, patients with high-TMB had longer median survival time compared low-TMB ones. Besides, higher TMB value was positively correlated with older age (Fig. 1F, P = 0.00099), male gender (Fig. 1G, P = 0.0035) and early M categories (Fig. 1H, P = 0.0078). Whereas, no significant correlation of TMB value was discovered with pathological grade, TNM staging, T status and M status (Additional file 2: Figures S2B-E).

### WGCNA co-expression network construction

**Fig. 2:**
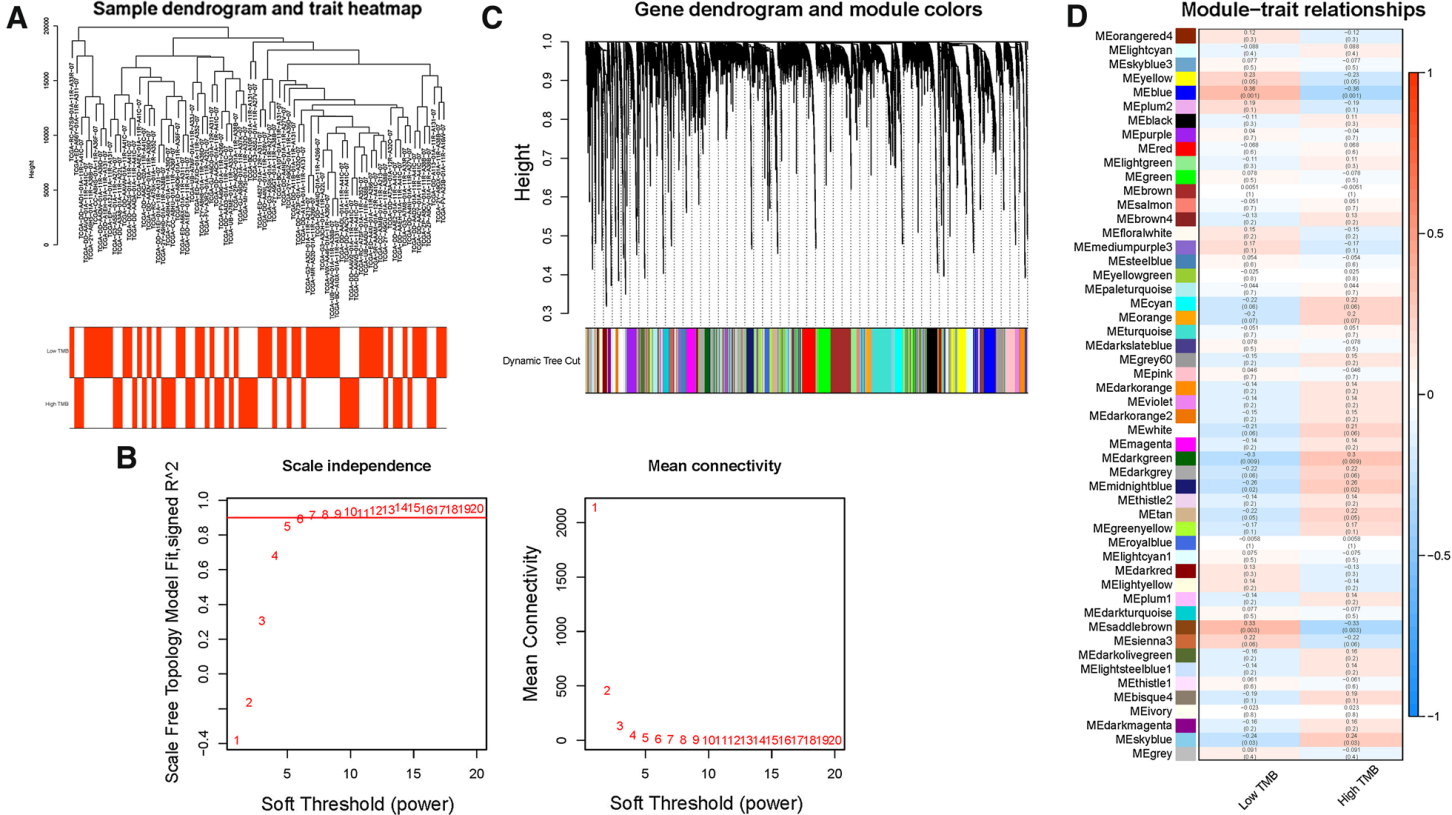
Construction of weighted gene co-expression network of HCC samples. **A** Sample dendrogram and clinical-traits heatmap was plotted. **B** Selection of the soft threshold made the index of scale-free topologies reach 0.90 and analysis of the average connectivity of 1–20 soft threshold power. **C** TMB-related genes with similar expression patterns were merged into the same module using a dynamic tree-cutting algorithm, creating a hierarchical clustering tree. **D** Heatmap of the correlations between the modules and TMB value (traits). Within every square, the number on the top refers to the coefficient between the TMB level and corresponding module, and the bottom is the P value

To identify TMB hub genes, WGCNA analysis was performed to construct the co-expression network for mRNA expression data of 17,932 genes together with TMB information. Sample dendrogram and TMB-traits heatmap were plotted (Fig. 2A). In order to construct the scaleless network, the optimal soft threshold power (β) was set as 6 since it was the first power value when the index of scale-free topologies achieve 0.90 (Fig. 2B). Genes with similar expression patterns were introduced into the same module by dynamic tree-cutting algorithm (module size = 60), making a hierarchical clustering tree with modules (Fig. 2C). The parameter was set as 0.25 to merge closely associated modules. Finally, a total of 52 modules were identified (Fig. 2D). Then, the Module eigengenes (MEs) indicated that the blue module clearly showed the highest association with TMB stratification (r = 0.36, p = 0.001; Fig. 2D). Therefore, the blue module with 939 genes (Additional file 1: Table S3) was employed for further analysis.

### Identification of DEGs

**Fig. 3:**
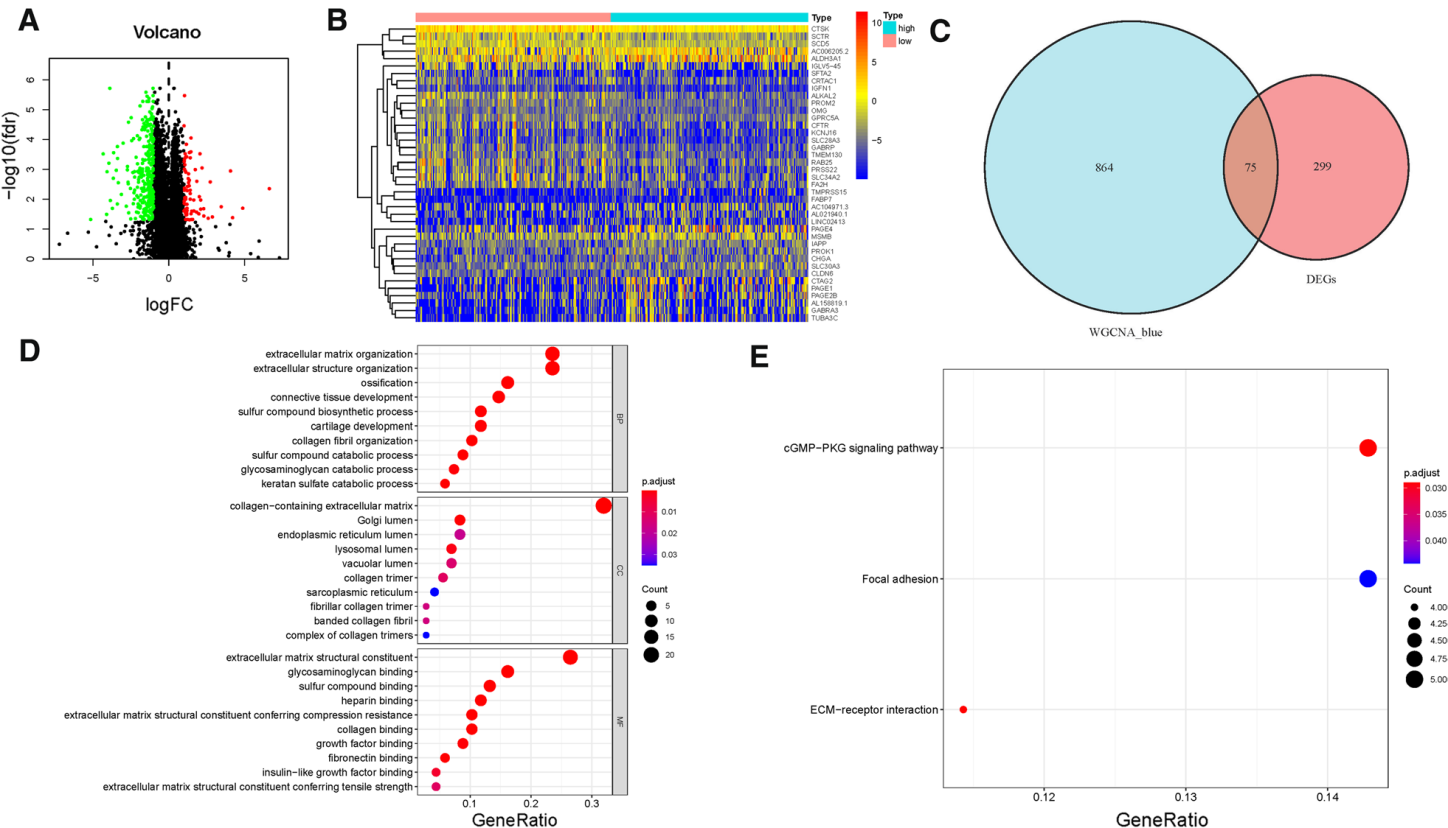
Differential analysis of gene expression data in high- and low-TMB groups and enrichment pathway annotation. **A** Volcano plot was delineated to visualize the DEGs. Red represented upregulated and green represented downregulated. **B** Heatmap of top 40 DEGs was drawn to reveal different distribution of expression state, where the colors of red to blue represented alterations from high expression to low expression. **C** Venn diagram of the hub genes from WGCNA blue module and DEGs. Pathway enrichment analyses of TMB hub genes. **D** Gene Ontology (GO) enrichment analysis of naïve B cells-related genes: biological processes (BP), cellular components (CC) and molecular function (MF). **E** KEGG enrichment analysis of naïve B cells-related genes

To further reveal the difference between low-/high-TMB groups from mRNA level, DEGs were analyzed. In total, 374 DEGs (300 down-regulated and 74 up-regulated) were determined as described previously (Fig. 3A, Additional file 1: Table S4). The heatmap presented the distribution of top 40 DEGs (Fig. 3B). A Venn diagram of TMB hub genes was plotted (Fig. 3C), uncovering 75 significant targets that overlapped between the blue module and DEGs.

### GO and KEGG functional annotation

To reveal the potential role of TMB hub genes in biological process, we conducted GO and KEGG annotation. The results of GO enrichment pathway analysis suggested that hub genes were mainly enriched in extracellular matrix organization, extracellular structure organization, ossification in biological processes (BP); collagen − containing extracellular matrix, Golgi lumen and endoplasmic reticulum lumen in cellular components (CC); extracellular matrix structural constituent, glycosaminoglycan binding, sulfur compound binding in molecular function (MF; Fig. 3D). For KEGG analysis, the top enriched terms were cGMP − PKG signaling pathway, Focal adhesion and ECM − receptor interaction (Fig. 3E). A detailed description is provided in Additional file 1: Table S5.

### Identification of TMB-based prognostic signature

To identify 75 hub genes with the most excellent prognostic performance, LASSO algorithm was employed (Additional file 2: Figure S3A, S3B). And three hub genes, including HTRA3, OLFM1 and PLN, were identified to yield prognostic signature to obtain risk score for HCC samples. The risk score was calculated: risk score = (0.0053 ∗ HTRA3 expression) + (0.0201 ∗ OLFM1 expression)—(0.0471 ∗ PLN expression). Then, HCC samples were assigned into low-/high-risk subgroups when setting the median value as cut-off point.

### Identification of TMB-based prognostic signature

**Fig. 4:**
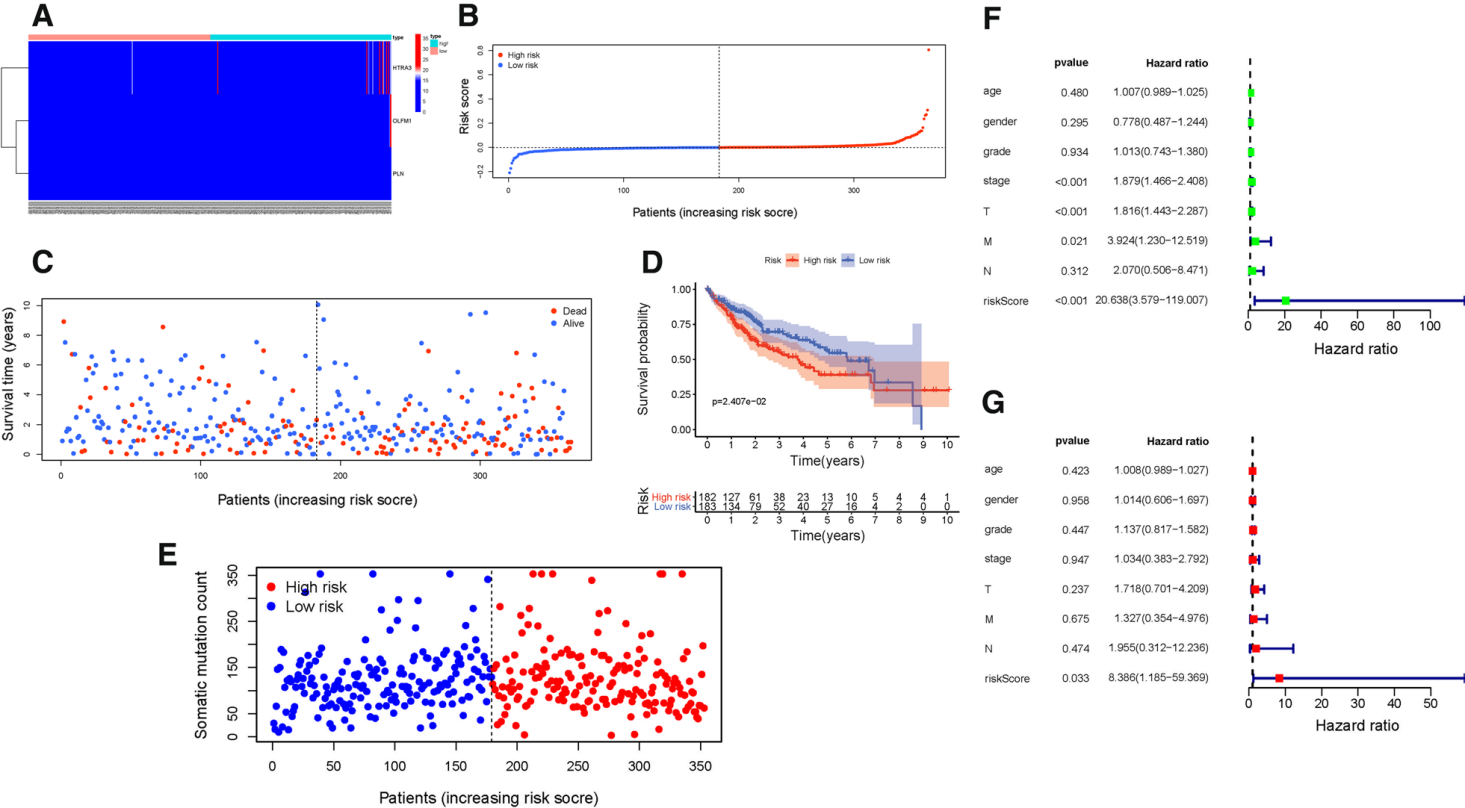
Validation of the prognostic risk signature in discovery group. **A** Heatmap presents the expression pattern of three hub genes in each patient. **B** Distribution of multi-genes signature risk score. **C** The survival status and interval of HCC patients. **D** Kaplan–Meier curve analysis presenting difference of overall survival between the high-risk and low-risk groups. **E** Distribution of somatic mutation count. **F** Univariate Cox regression analyses of overall survival. **G** Multivariate Cox regression analyses of overall survival

The distributions of hub genes expression value with corresponding subgroups and patients were delineated in Fig. 4A. The allocations of risk score and dot pot of survival status indicated that HCC samples with high-risk exhibited poorer prognosis (Figs. 4B and C). Additionally, K–M survival analysis supported that low-risk patients had significant higher overall survival rate (P = 2.407e−02; Fig. 4D). The distributions of samples with corresponding risk score and somatic mutation count were presented in Fig. 4E.Furthermore, univariate Cox regression analysis presented the hazard ratio (HR) of risk score was 20.638 (95% CI: 3.579 − 119.007; Fig. 4F). And multivariate Cox regression showed corresponding results (HR = 8.386, 95% CI: 1.185 − 59.369; Fig. 4G), supporting risk score was an independent prognostic factor.

### Validation of TMB-based prognostic signature

To explore prognostic validity of risk score, above findings were confirmed in the external validation cohort. The according results displayed the distributions of hub genes, risk score, and survival status in the external validation cohort (Additional file 2: Figure S4A, S4B and S4C). Consistent with the results in discovery set, K–M curves presented that high-risk HCC patients had shorter overall survival time, though there was no statistical difference (Additional file 2: Figure S4D). Additionally, ROC curves were plotted and AUC value for the 3-year OS reached 0.62, suggesting great predictive accuracy (Additional file 2: Figure S4E).

### Clinical significance of risk score

**Fig. 5:**
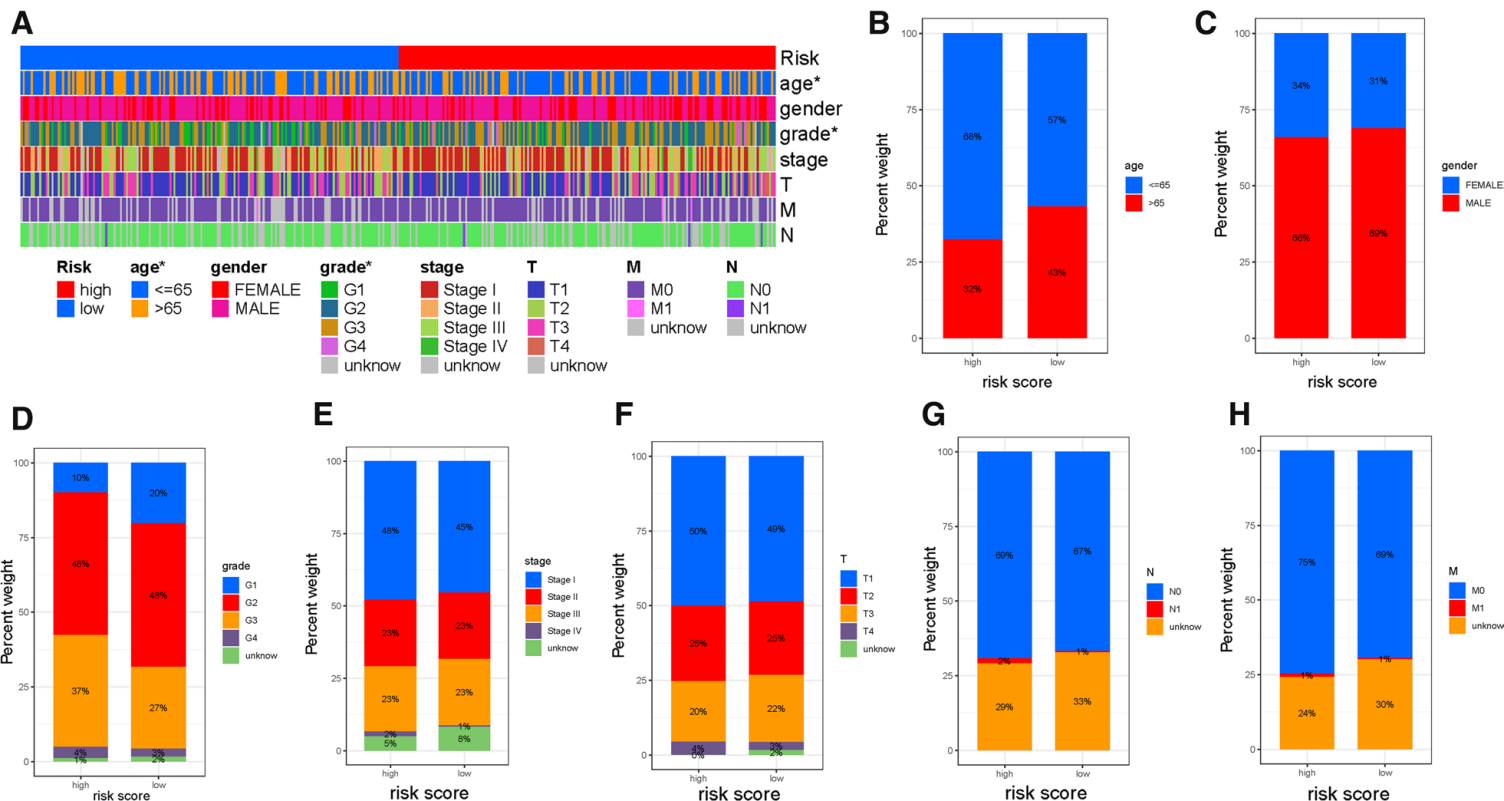
Clinical significance of the prognostic risk signature. **A** Heatmap presents the distribution of clinical feature and corresponding risk score in each sample. Rate of clinical variables subtypes in high or low risk score groups. **B** Age, **C** Gender, **D** WHO grade, **E** clinical stage, **F** T status, **G** N status and **H** M status

Firstly, the distribution of clinicopathological features subtypes in different risk groups was explored and visualized (Fig. 5A). Figure 5B–H showed that fraction of subtypes according to age, gender, pathological grade, clinical stage, T category, N status and M status in high-/low-risk group, respectively. Furthermore, to confirm whether prognostic signature remained robust prognosis prediction validity in patients subdivided into different subtypes according to clinicopathological variables, stratification analysis was performed. Compared with low-risk samples, HCC patients with high-risk had lower overall survival rate in both the young (< = 65) and old (> 65) groups (Additional file 2: Figures S5A and S5B). Likewise, risk score suggested prognostic difference well for samples in female gender or male gender (Additional file 2: Figures S5C and S5D), grade 1–2 or G3-4 category (Additional file 2: Figures S5E and S5F), samples with early- or late-stage (Additional file 2: Figures S5G and S5H), samples in T1-2 or T3-4 category (Additional file 2: Figures S5I and S5J), samples in N0 status (Additional file 2: Figures S5K) and samples in M0 category (Additional file 2: Figures S5L). These results demonstrated that risk score was an outstanding prognostic predictor.

### Development of prognostic nomogram

**Fig. 6:**
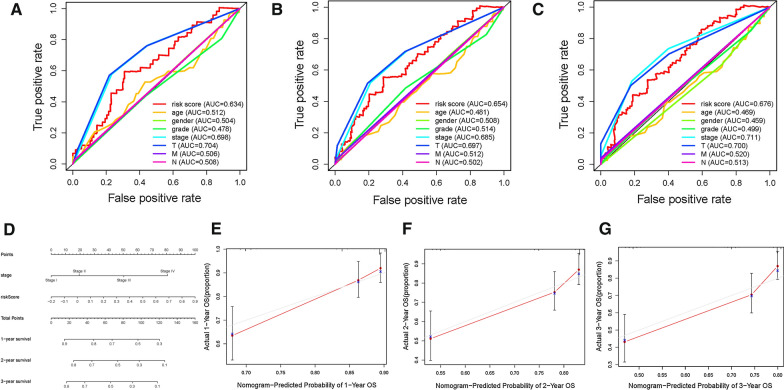
Validation of prognostic efficiency of risk score in HCC. (A-C) Areas under curves (AUCs) of the risk scores for predicting 4-, 5-, and 6-year overall survival time with other clinical characteristics. **D** Nomogram was assembled by age and risk signature for predicting survival of HCC patients. **E** One‐year nomogram calibration curves. **F** Two‐year nomogram calibration curves. **G** Three‐year nomogram calibration curves

To demonstrate risk score was the best prognostic predictor, age, gender, clinical stage and tumor grade were listed as the candidate indicators. These clinical variables were introduced into the AUC analysis for 1-, 2-, 3-, 4-, 5-, and 6-year OS and risk signature were found to obtain the most AUC value (Additional file 2: Figures S6A, S6B, S6C, Fig. 6A–C). Then a prognostic nomogram including risk score and clinical stage was delineated to predict overall survival rate quantitatively (Fig. 6D). Age, gender and tumor grade were excluded out of the nomogram given their AUCs did not reach 0.6. Calibrate curves was plotted to support great prognostic predictive validity of overall survival rate in as-constructed nomogram (Figs. 6E–G).

### Role of risk score in TIME context

**Fig. 7:**
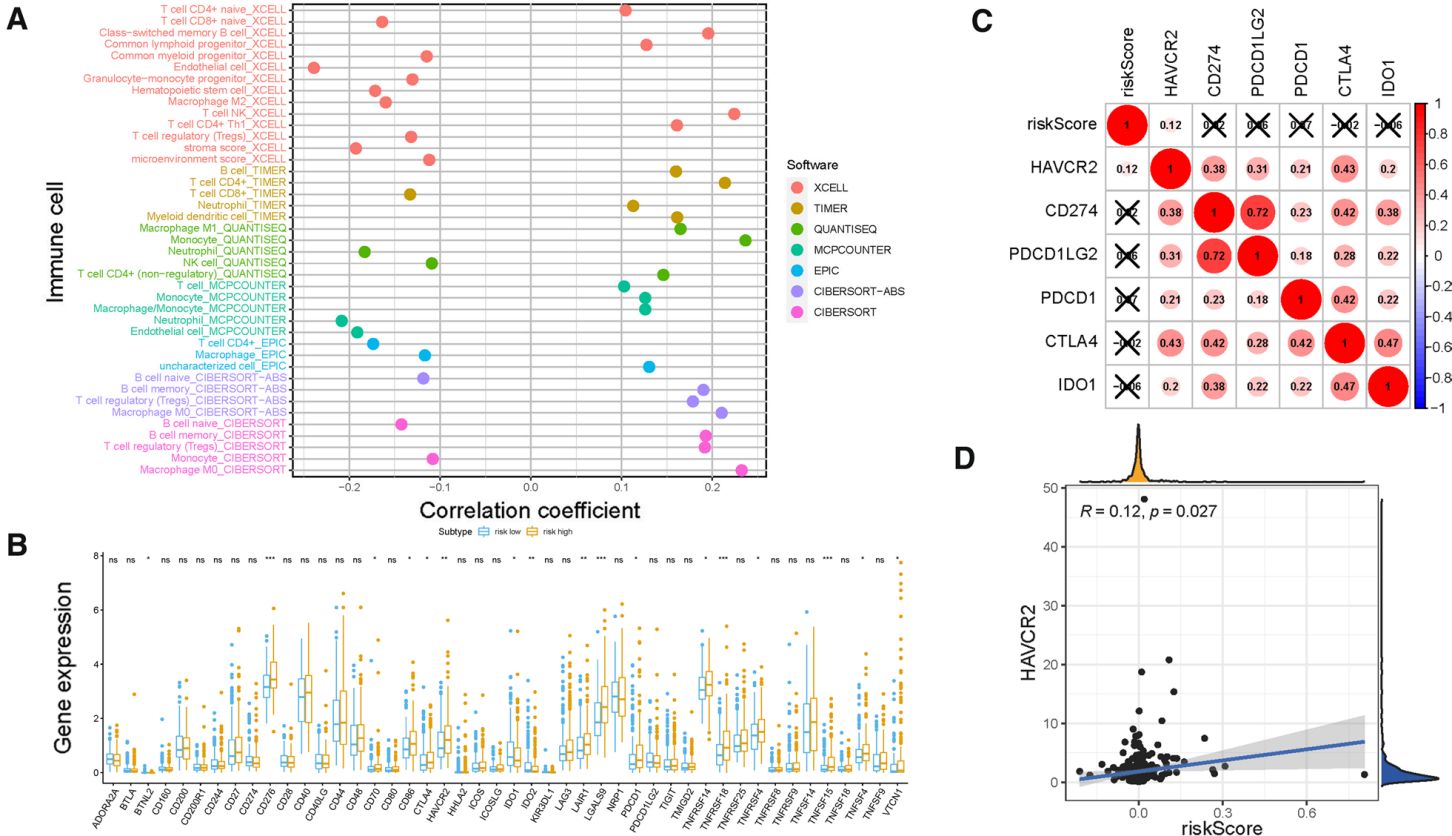
Estimation of Tumor-Infiltrating Cells and Immunotherapy significance. **A** Patients in the high-risk group were more positively associated with tumor-infiltrating immune cells, as shown by Spearman correlation analysis. Correlation between prognostic risk signature with hub immune checkpoint genes. **B** Correlation analysis between immune checkpoint inhibitors (CD274, PDCD1, PDCD1LG2, CTLA4, HAVCR2, and IDO1) with prognostic risk signature. **C** Correlation between prognostic risk signature and HAVCR2

Existing studies have contributed strong evidence to demonstrate that high tumor burden mutation (TMB) was correlated with increasement of infiltrating CD8 + T cells, which recognized tumor neoantigens then resulted in intense tumor-killing effects to annihilate tumor cells [19, 21, 33].Thus, the potential contribution of TMB-based signature in diversity and complexity of TIME was further explored. The results showed that high risk score was significantly and negatively correlated with abundance of Neutrophil, naïve B cells and Endothelial cell, whereas positively related with infiltration of Memory B cells, M0 Macrophages and Monocytes (Additional file 2: Figures S7, S8). Furthermore, Spearman correlation analysis was further performed (Fig. 7A) and the detailed results were provided in Additional file 1: Table S6. These findings suggested that low-risk group characteristic with immune response activated condition, which may contribute to anti-tumor effect.

Additionally, 17 of 47 (i.e., CTLA‐4, etc.,) ICB-associated targets correlated significantly with risk score (Fig. 7B). These findings suggested that risk score may act as nonnegligible player in regulation of immune response further immunotherapeutic efficacy. Besides, the potential function of risk score in immunotherapy was further explored. First, the correlation of immunotherapy key targets (PDCD1, CD274, PDCD1LG2, CTLA‐4, HAVCR2, and IDO1) [28–30] with risk score was performed (Fig. 7C). And risk score was positively and significantly correlated with PDCD1 (r = 0.12; P = 0.027; Figs. 7D), indicating risk score might serve as a pivotal player in the prediction of clinical outcome of immunotherapy in HCC.

### HTRA3 significantly affected overall survival and correlates with immune infiltration ICB vital targets

**Fig. 8:**
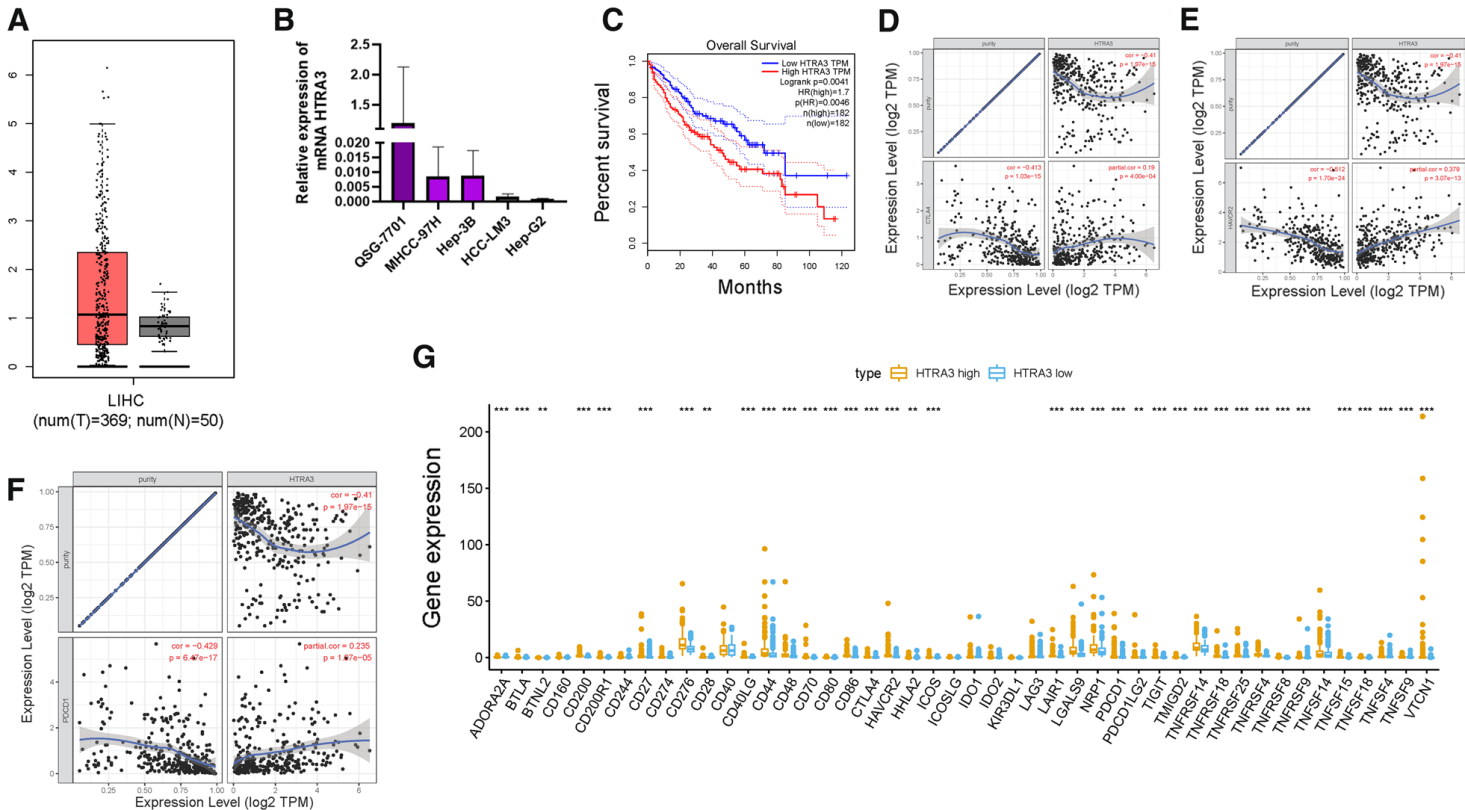
The role of HTRA3 in prognosis and immunotherapy of HCC. HTRA3 are upregulated in HCC samples based on TCGA dataset (**A**) and experimental validation (**B**), and lower HTRA3 expression level was significantly correlated with longer overall survival time (**C**). The association between the expression levels of HTRA3 with CTLA4 (**D**), HAVCR2 (**E**), and PDCD1 (**F**) using TIMER database. **G** Comparison of immune checkpoint blockade-related genes expression levels between low-HTRA3 group and high-HTRA3 groups

HTRA3, considered as beneficial indicator in this risk signature, had not been explored in HCC. Thus, the underlying role of HTRA3 in HCC was validated in further experiments. Firstly, the expression level of HTRA3 between normal hepatic samples and tumor tissues was analyzed according to TCGA database. GEPIA website was employed to validate the expression levels of HTRA3 [34]. The GEPIA results showed that there was no significant difference of HTRA3 expression between two different samples (Fig. 8A). Taking advantage of qRT-PCR, expression level of HTRA3 was detected in four different HCC cell lines (MHCC-97H, Hep-3B, HCC-LM3, HepG2) and normal liver cell line (SQG-7701). HTRA3 was downregulated in tumor cells compared with normal counterpart (Fig. 8B). To further assess prognostic performance of HTRA3, K-M survival curve was plotted based on samples assigned into HTRA3 low-and high-expressed subgroups. The result presented that samples with lower HTRA3 expression exhibited significant overall survival rate advantage (Fig. 8C, P = 0.0041).

To reveal the potential role of HTRA3 in immunotherapy, the correlation between expression level of HTRA3 with expression level of immunotherapy hub targets adjusted by tumor purity was analyzed using TIMER database. TIMER results shown that HTRA3 presented significant positive correlation with CTLA4 (r = 0.19; P = 4.00e−04), HAVCR2 (r = 0.379; P = 3.07e−13), and PDCD1(r = 0.235; P = 1.07e−05; Figs. 8D–F). According to correlation analysis, 36 of 47 ICB-related genes (i.e., PDCD1, CTLA4, etc.,) expression levels were remarkably higher in subjects with high-HTRA3 relative to low-HTRA3 ones (Fig. 8G), suggesting vital role of HTRA3 in immunotherapy.

### Genes mutation in risk score

**Fig. 9:**
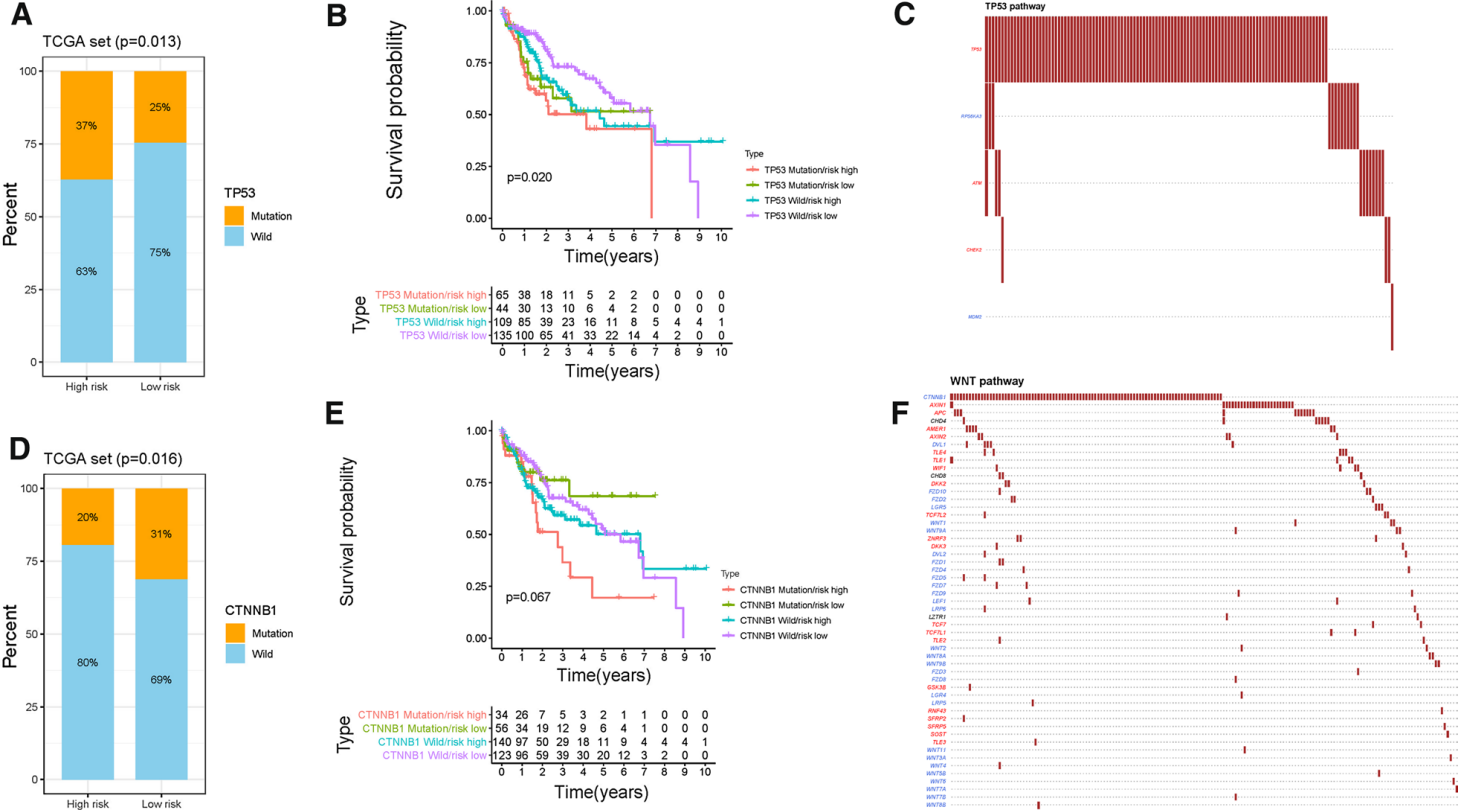
Correlation of mutation of genes with risk score. **A** The proportion of mutation of TP53 between two risk score subgroups. **B** Kaplan–Meier curves for patients stratified by both mutation of TP53 and risk score. **C** Mutation data of TP53-relevant pathway. **E** The proportion of mutation of CTNNB1 between two risk score subgroups. **F** Kaplan–Meier curves for patients stratified by both mutation of CTNNB1 and risk score. **G** Mutation data of CTNNB1-relevant pathway

According to results of somatic mutation data, TP53, TTN and CTNNB1 were the top 3 genes with highest mutation frequency (Fig. 1A). Thus, the potential role of genes mutation was uncovered in risk score and the proportion of mutation gene was analyzed in both high- and low-risk groups. Besides, mutation of TP53 was significantly and positively correlated with risk score (Fig. 9A),whereas mutation of CTNNB1 exhibited opposing trend and mutation of TTN without significantly correlation with risk score (Figs. 9D and Additional file 2: Figure S9A). Next, the synergistic effect of risk score and gene mutation was estimated in prognostic stratification. Stratified survival analysis suggested that the mutation of TP53 did not interfere with risk scores-based predictions. Risk score subgroups presented significant prognosis differences in both TP53 mutation and TP53 wild subgroups (Fig. 9B). However, risk score was not prognostic indicator independent of TTN and CTNNB1 mutation (Fig. 9E and Additional file 2: Figure S9B). To elucidate the cumulative effect of mutated gene-relevant pathway in somatic mutation, the mutation status of genes downstream targets was analyzed (Figs. 9C and F, TP53 and CTTNB1, respectively). The result showed that mutation of genes (TP53 and CTTNB1) was predominant among relevant pathways targets.

## Discussion

HCC is the one of most aggressive and lethal malignancies, well characterized with high morbidity globally [1–3]. It’s well established that such genetic alternation as TP53 mutation, alternative splicing, DNA methylation and regulation of non-coding RNA acted as a pivotal player in HCC development [4, 35–38]. By now, more and more studies have been performed to reveal the potential role of infiltrating immune cells in cancer progression, including HCC [39, 40]. Immunotherapy, which facilitated the immune cells to eliminate tumor cells, has exhibited promising therapeutic efficiency and encouraging clinical outcome in anti-tumor treatment [41, 42]. Clinical studies pointed out that administration of immune checkpoint inhibitors in advanced HCC have shown benefits, however, just approximately one fifth patients responded to immunotherapy [17]. Thus, it is of great importance predicting therapeutic outcome to optimize treatment benefit and personalized tailored therapy.

Recently, TMB has been identified as an effective and novel indicator to predict response to immunotherapy in a variety of malignancies [43–45]. However, the correlation of TMB status hub genes with prognostic prediction, immune infiltration and immunotherapeutic result in HCC is still unclear. Hence, this study was designed to determine TMB status hub genes and pivotal biological processes, further yield a prognostic signature and potential target for immune microenvironment landscape depiction and precision immunotherapy prediction.

Herein, this research was designed to elucidate the potential significance of TMB related molecules in HCC. Firstly, landscape of somatic mutation was analyzed and delineated. TMB level was demonstrated to be correlated with clinical variables (age, gender and T status). Then, differentially expressed genes (DEGs) between low and high TMB level subgroups were analyzed to further identification TMB candidate genes coordinated with WGCNA co-express network. The results of subsequent enrichment pathway analysis presented that hub genes were mainly enriched in extracellular matrix structural related pathways and cGMP − PKG signaling pathway.

With the help of LASSO regression analysis, candidate genes were further determined and final prognostic signature including HTRA3, OLFM1 and PLN was established.

To validate great prognostic accuracy, survival analysis and ROC curve were performed in both discovery group and external validation cohort. Furthermore, risk score was demonstrated to be an independent prognostic factor using both univariable and multivariable regression analysis. Additionally, prognostic nomogram was constructed to facilitate extension and popularization. Furthermore, the correlation of risk score with clinical variables was analyzed and risk score was demonstrated to retain excellent prognostic performance when HCC cases divided into groups based on clinicopathological factors.

Given risk signature derived from TMB, which was significantly correlated with immune surveillance, the potential role of risk score in complexity of TIME and immunotherapeutic effect was further investigated. The results pointed out that risk score was negatively related with activated immune cell (i.e., Neutrophil, etc.,), implying low risk score patients was immune activated phenotype, in line with higher risk score suggested shorter overall survival. Furthermore, risk score was significantly and positively correlated with the immunotherapeutic hub targets (i.e., HAVCR2, etc.,), suggesting samples with high-risk score might be more affected by immune checkpoint blockade pathways, then inhibited anti-tumor immune activation and deteriorate prognosis accordingly. Since no immunotherapy data in HCC cohort, it was unable to further explore the correlation of risk score with response of immunotherapy.

High temperature requirement A3 (HTRA3), a member of the HtrA family, has been reported as a cancer antagonist in cancer progression of multiple tumor types [46–48]. Currently, yet its molecular functions of HTRA3 in HCC are not well understood. Thus, this study attempted to explore the prognostic predictive significance of HTRA3 in immunotherapy of HCC. The result of qRT-PCR showed that HTRA3 expression level is significantly downregulated in HCC cells. However, low expression of HTRA3 suggested better prognosis according to TCGA database. Additionally, HTRA3 expression level was positively correlated with most immune checkpoint blockade pathway targets. Collectively, high-HTRA3 samples presented immunosuppressive condition thus facilitate tumor immune evasion, leading to poor overall survival rate accordingly. Nevertheless, the biological role of HTRA3 in HCC remains lacking, which needs further and deeper experimental exploration.

To elucidate the cumulative effective of mutation genes, top 3 genes with highest mutation frequency were selected for further analysis. Notably, mutation of TP53 was significantly and positively correlated with risk score. Furthermore, the prognosis value of risk score was independent of mutation of TP53. In the TP53-relevant pathway, gene mutation was mainly enriched in mutation of TP53.

Compared with published articles that investigated the TMB status in HCC, it was worthy mentioned that there were some superiorities in this study. Firstly, all HCC cases from TCGA-LIHC project and ICGC-LIRI-JP dataset were included for thoroughly analysis, and the total specimen size was considerably large. Furthermore, WGCNA network and DEGs analysis were integrated to comprehensively identify difference between low-/high-TMB subgroups from sequencing level. Additionally, seven algorithms (XCELL, TIMER, QUANTISEQ, MCPcounter, EPIC, CIBERSORT, and CIBERSORT-ABS) were performed to elucidate the potential players of TMB hub genes in the formation of TIME complexity and diversity and immunotherapeutic outcome. Besides, as we know, this work is the first placing emphasis on the biological role of HRTA3 and cumulative effect of TP53 mutation in HCC.

## Conclusion

In conclusion, systematical bioinformatic analyses in prognosis predictive value of TMB were performed, which was proposed to improve prognosis prediction in HCC. Moreover, a robust and promising prognostic clinical-risk nomogram with encouraging potential for clinical practice was constructed to predict clinical outcome quantitatively. It is noteworthy that the comprehensive analysis of TMB status hub genes in the context of TIME will facilitate understanding TMB from biological standpoint and contribute into tailored immunotherapeutic administration. Notwithstanding, these results required further experimental and more clinical exploration focusing on tumor initiation and development and the roles of TMB status hub genes in HCC.

## Supplementary Information

**Additional file 1.** **Supplementary Table S1**: Clinical Characteristics of the HCC patients in TCGA and ICGC.**Supplementary Table S2**: TMB value of 365 HCC patients. **Supplementary Table S3**: The genes and corresponding modules after WGCNA. **Supplementary Table S4**: differentially expressed genes (DEGs) between low- and high-TMB groups. **Supplementary Table S5**: The Functional Annotation analysis of hub genes. **Supplementary Table S6**: The results of correlation of risk score with immune infiltrating cell.

**Additional file 2.** **Supplementary Figure S1**. Overall research design. Flow-process diagram presenting the process of comprehensive analysis. **Supplementary Figure S2**: Prognostic analysis of TMB and correlation with clinical characteristics. (**A**) Higher TMB levels correlated with better survival outcomes though P>0.05. (**B**–**E**) No significant difference of TMB levels was observed with clinical grade, AJCC stage, T status and M status. **Supplementary Figure S3**: Regression coefficient diagram based on LASSO algorithm. (**A**) LASSO coefficient profiles of 75 hub genes. A vertical line is drawn at the value chosen by 10‐fold cross‐validation. (**B**) Ten‐time cross‐validation for tuning parameter selection in the lasso regression. The vertical lines are plotted based on the optimal data according to the minimum criteria and 1-standard error criterion. The left vertical line represents the 3 hub genes finally identified. **Supplementary Figure S4**: Confirmation of risk score in the external validation group. (**A**) Heatmap presents the expression pattern of three hub genes in each patient. (**B**) Distribution of multi-genes signature risk score. (**C**) The survival status and interval of HCC patients. (**D**) Kaplan–Meier curve analysis presenting difference of overall survival between the high-risk and lowrisk groups. (**E**) ROC analysis was employed to estimate the prediction value of the prognostic signature. **Supplementary Figure S5**: Kaplan–Meier survival analysis for multiple HCC subgroups stratified by clinical variables. (**A**, **B**) Age. (**C**, **D**) Gender. (**E**, **F**) Tumor grade. (**G**,**H**) Stage. (**I**, **J**) T status. (**K**) N status. (**L**) M status. **Supplementary Figure S6**: (**A**-**C**) Areas under curves (AUCs) of the risk scores for predicting 1-, 2-, and 3-year overall survival time with other clinical characteristics. **Supplementary Figure S7-S8**: The representative results ofthe evaluation of tumor infiltrating immune cells with risk signature. **Supplementary Figure S9**: Correlation of prognostic risk score with mutation of genes. (**A**) The proportion of mutation of TTN between two risk score subgroups. (**B**) Kaplan-Meier curves for patients stratified by both mutation of TTN and risk score.

## Data Availability

The datasets generated for this study can be found in the TCGA database (https://portal.gdc.cancer.gov) and ICGC database (https://dcc.icgc.org).

## Abbreviations

AUC: Area under the curve
BP: Biological processes
CC: Cellular components
CD274: Also known as PD-L1
CI: Confidence Interval
CTLA‐4: Cytotoxic T-lymphocyte-associated antigen 4
DCs: Dendritic cells
DEG: Differentially expressed genes
DEL: Deletion
FDR: False discovery rate
GO: Gene ontology
HCC: Hepatocellular carcinoma
HR: Hazard ratio
ICB: Immune checkpoint blockade
IDO1: Indoleamine 2,3-dioxygenase 1
INS: Insertion
KEGG: Kyoto Encyclopedia of Genes and Genomes
K-W: Kruskal–Wallis
LASSO: Least absolute shrinkage and selection operator
MAF: Mutation Annotation Format
MEs: Module eigengenes
MF: Molecular function
OS: Overall survival
PDCD1: Also known as PD-1
PDCD1LG2: Also known as PD‐L2
qRT-PCR: Quantitative real-time polymerase chain reaction
ROC: Receiver operating characteristic
SNP: Single nucleotide polymorphism
TCGA: The Cancer Genome Atlas
TICs: Tumor‐infiltrating immune cells
TILs: Tumor Infiltrating Lymphocytes
TIM-3: T cell immunoglobulin and mucin-domain containing-3
HAVCR2: Also known as
TIME: Tumor immune microenvironment
TMB: Tumor mutation burden
TNM: Tumor Node Metastasis
TOM: Topological overlap matrix
Tregs: Regulatory T cells
WGCNA: Weighted gene co-expression network analysis
