# Supplementary material for: Multi-omics analysis reveals prognostic value of tumor mutation burden in hepatocellular carcinoma
Source: Cancer Cell Int. 2021 Jul 3;21:342. doi: 10.1186/s12935-021-02049-w (PMC8254981; doi:10.1186/s12935-021-02049-w)

**Supplementary Figure Legends:**

**Figure S1.** Overall research design. Flow-process diagram presenting the process of comprehensive analysis.

**
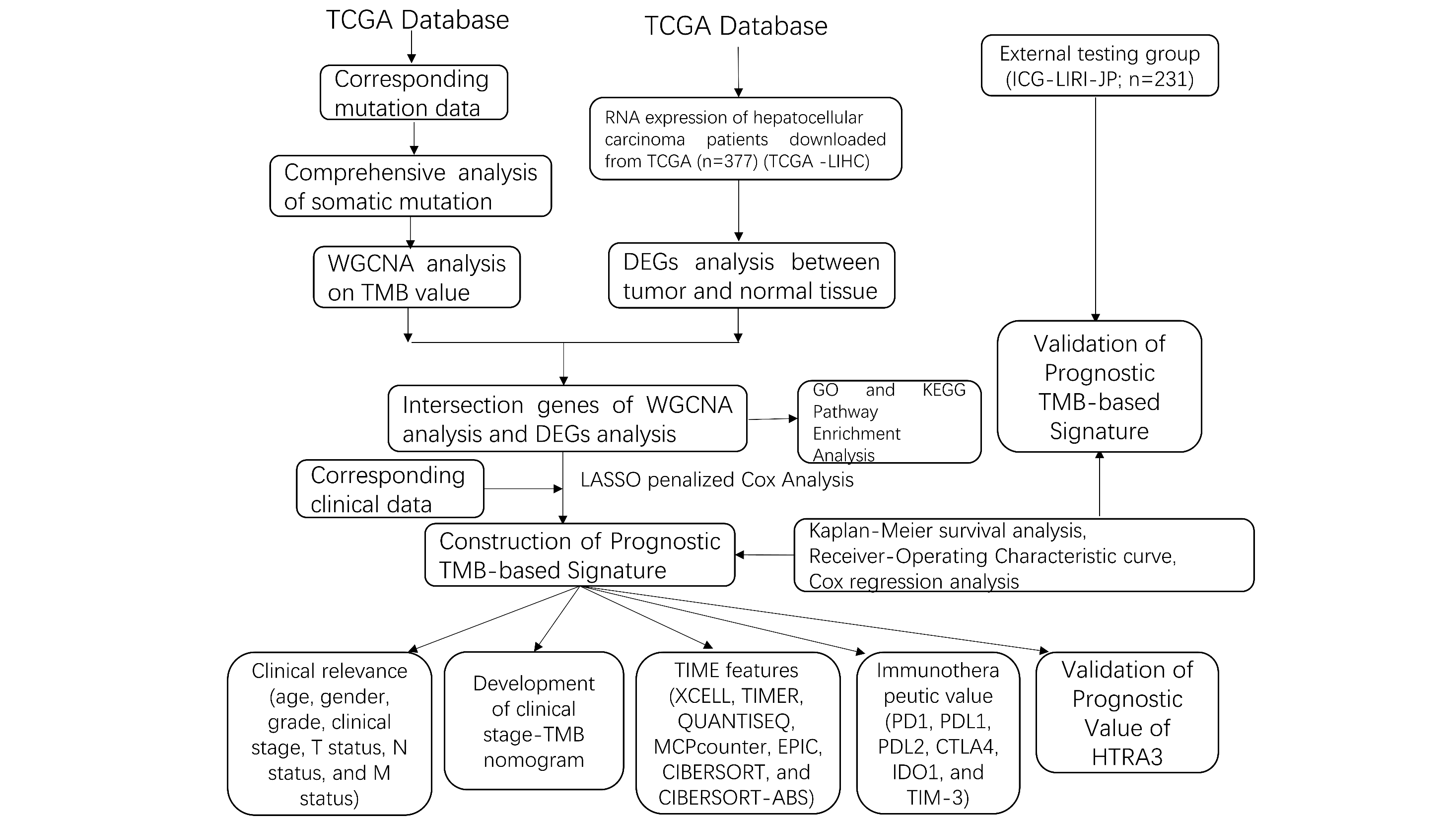
Figure S2:** **Prognostic analysis of TMB and correlation with clinical characteristics.** (A) Higher TMB levels correlated with better survival outcomes though P>0.05. (B-E) No significant difference of TMB levels was observed with clinical grade, AJCC stage, T status and M status.

**
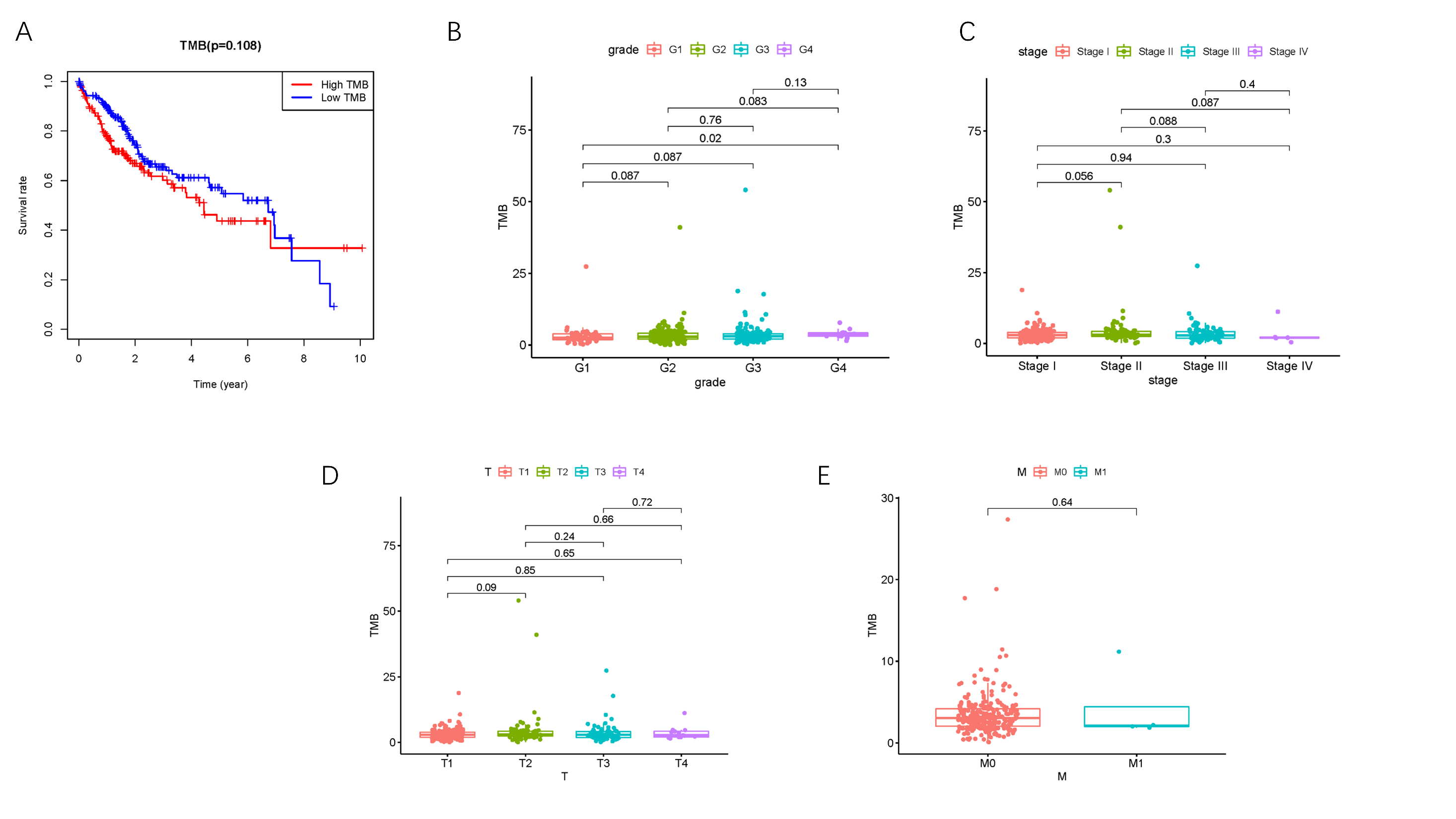
Figure S3: Regression coefficient diagram based on LASSO algorithm.** (A) LASSO coefficient profiles of 75 hub genes. A vertical line is drawn at the value chosen by 10‐fold cross‐validation. (B) Ten‐time cross‐validation for tuning parameter selection in the lasso regression. The vertical lines are plotted based on the optimal data according to the minimum criteria and 1-standard error criterion. The left vertical line represents the 3 hub genes finally identified.

**
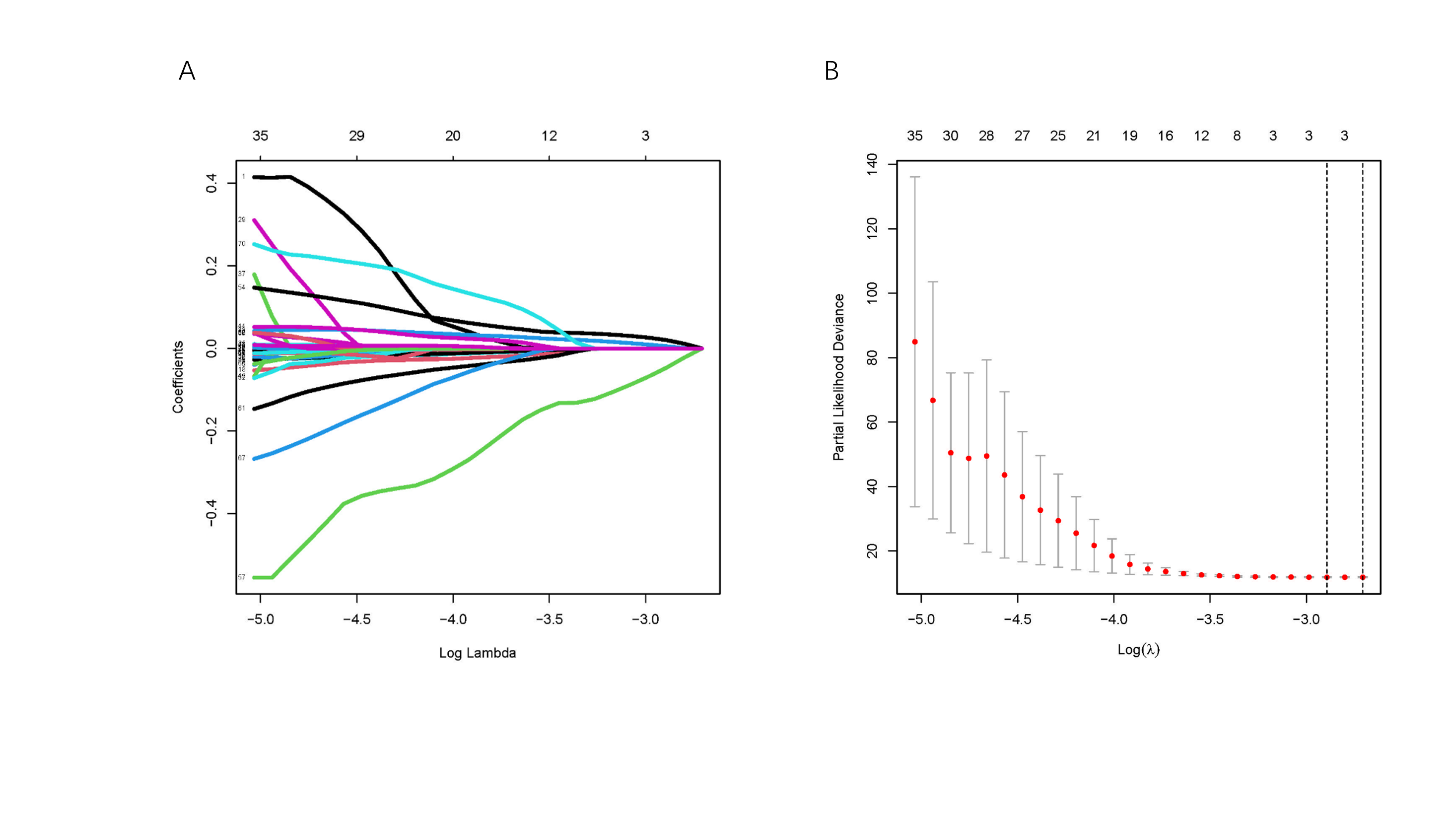
Figure S4: Confirmation of risk score in the external validation group.** (A) Heatmap presents the expression pattern of three hub genes in each patient. (B) Distribution of multi-genes signature risk score. (C) The survival status and interval of HCC patients. (D) Kaplan–Meier curve analysis presenting difference of overall survival between the high-risk and low-risk groups. (E) ROC analysis was employed to estimate the prediction value of the prognostic signature.

**
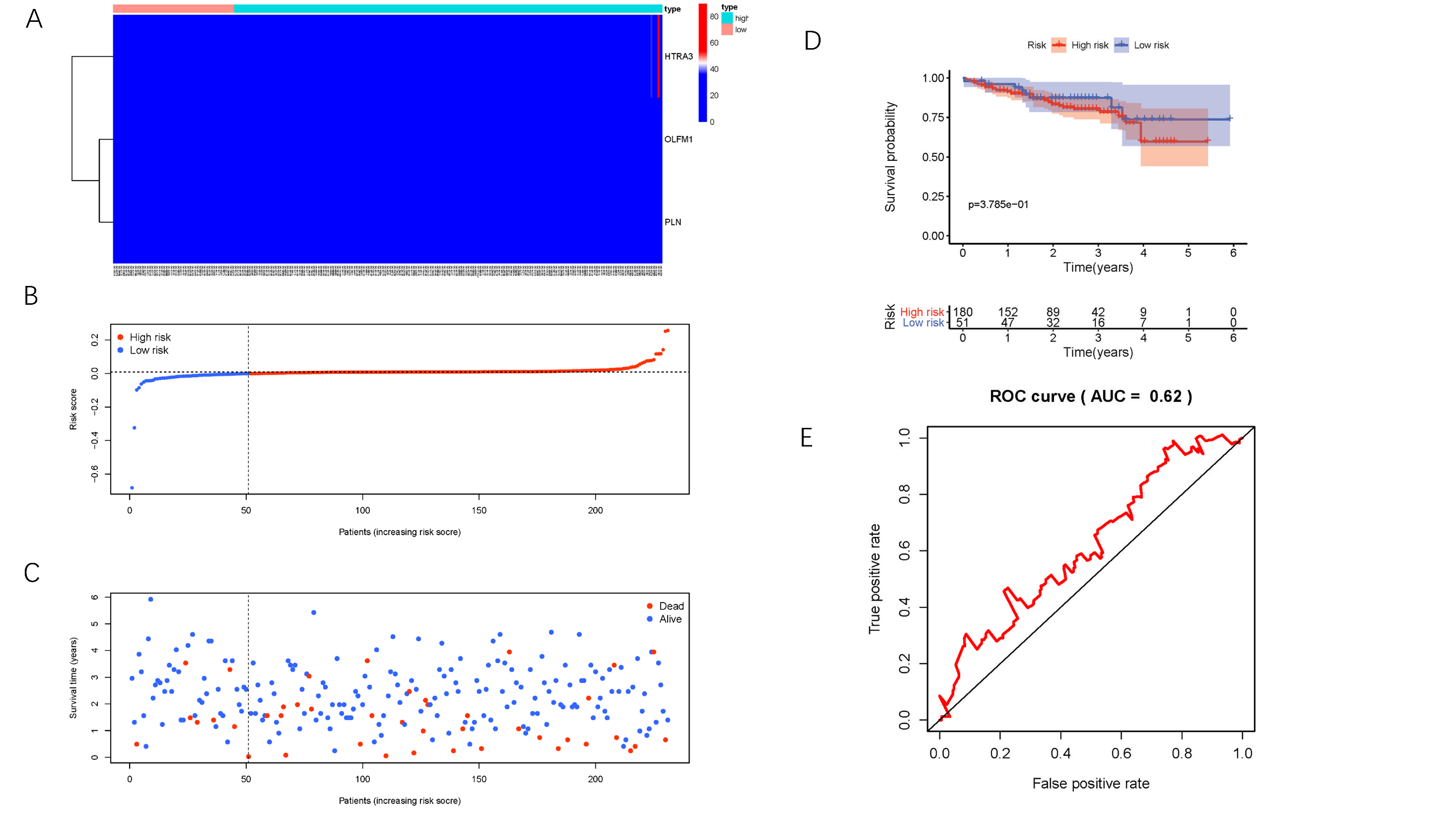
Figure S5: Kaplan–Meier survival analysis for multiple HCC subgroups stratified by clinical variables.** (A-B) Age. (C-D) Gender. (E-F) Tumor grade. (G-H) Stage. (I-J) T status. (K) N status. (L) M status.

**
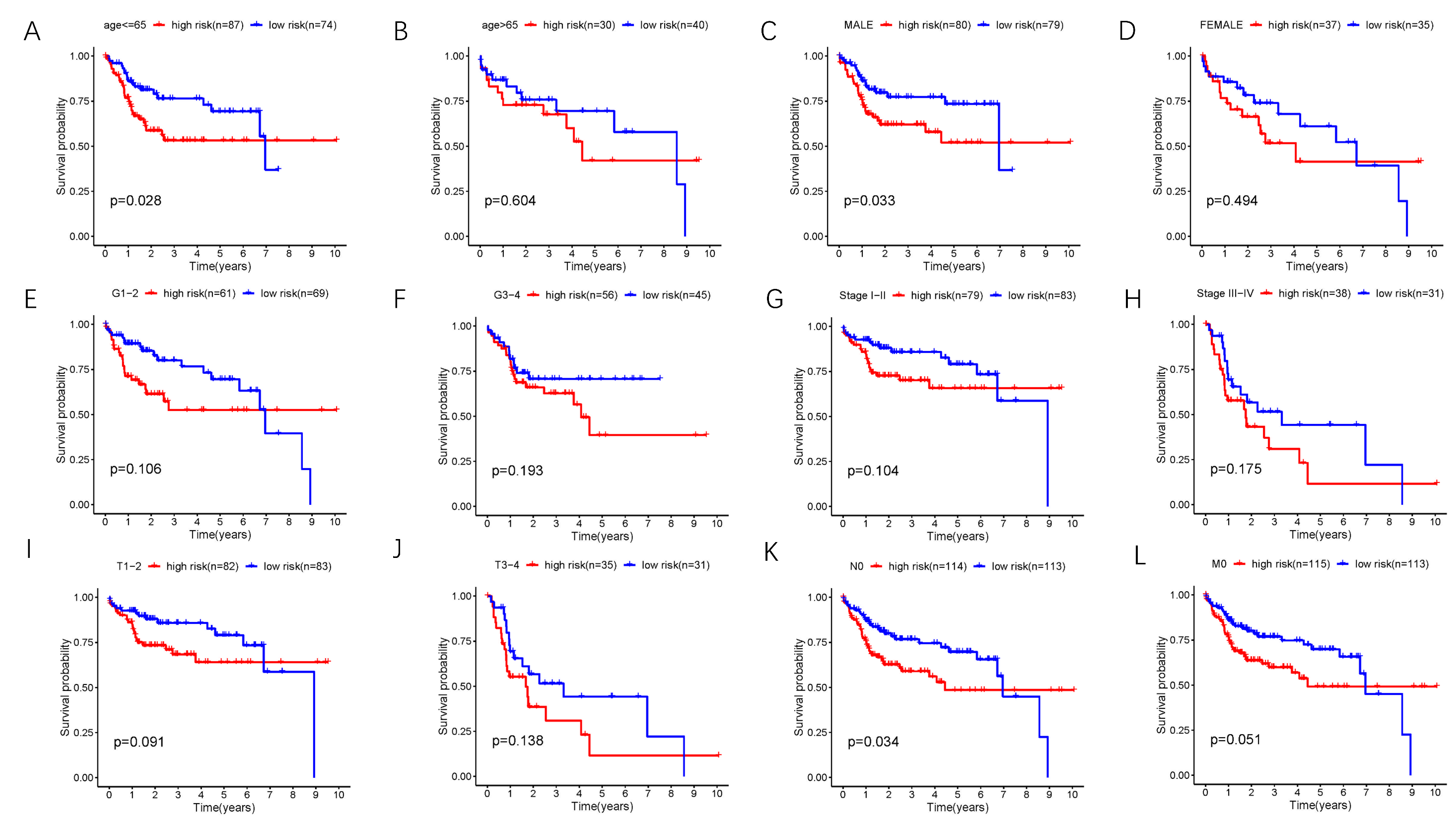
Figure S6:** (A-C) Areas under curves (AUCs) of the risk scores for predicting 1-, 2-, and 3-year overall survival time with other clinical characteristics.

**
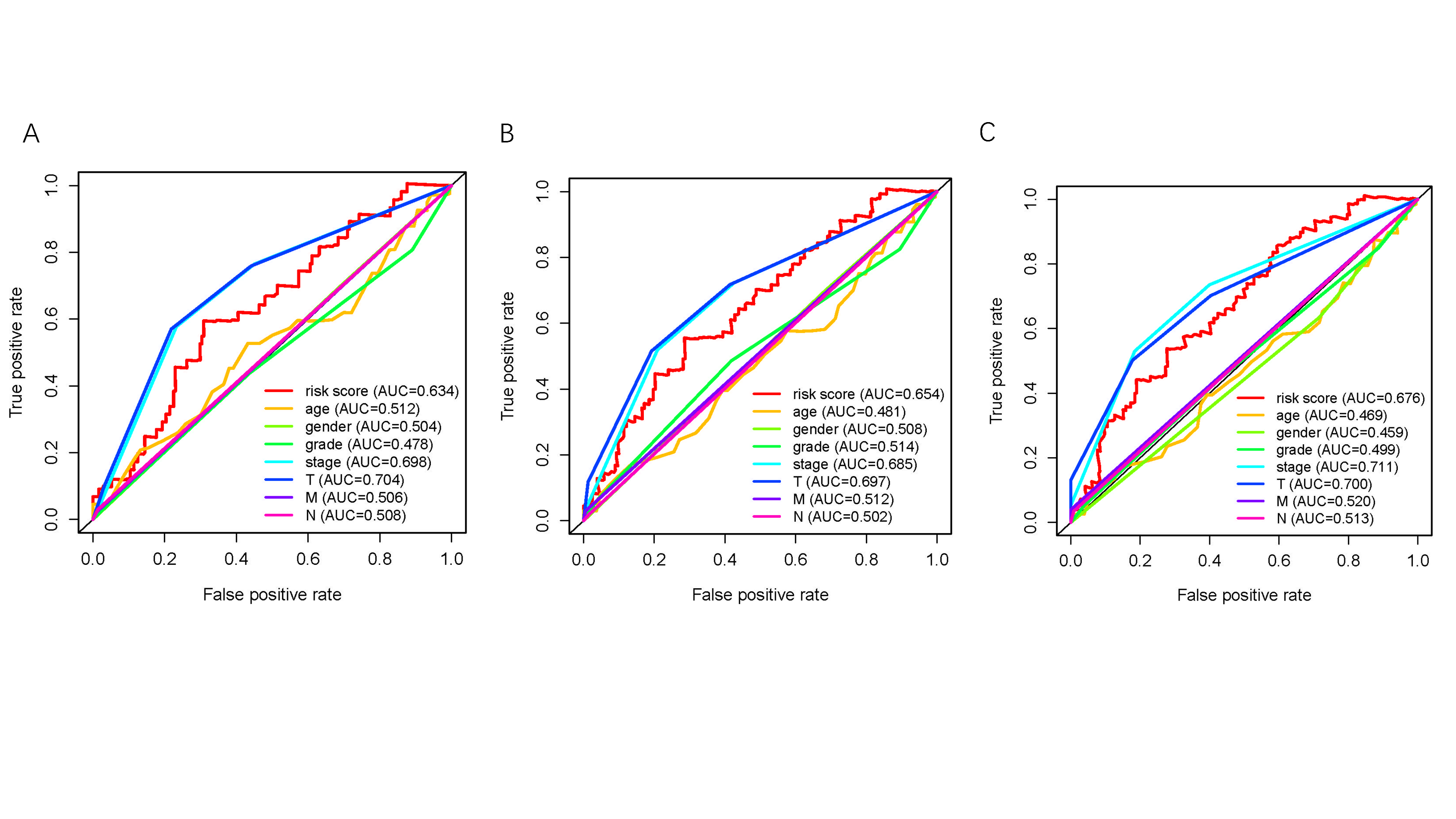
Figure S7-S8: The representative results of the evaluation of tumor infiltrating immune cells with risk signature.**

**
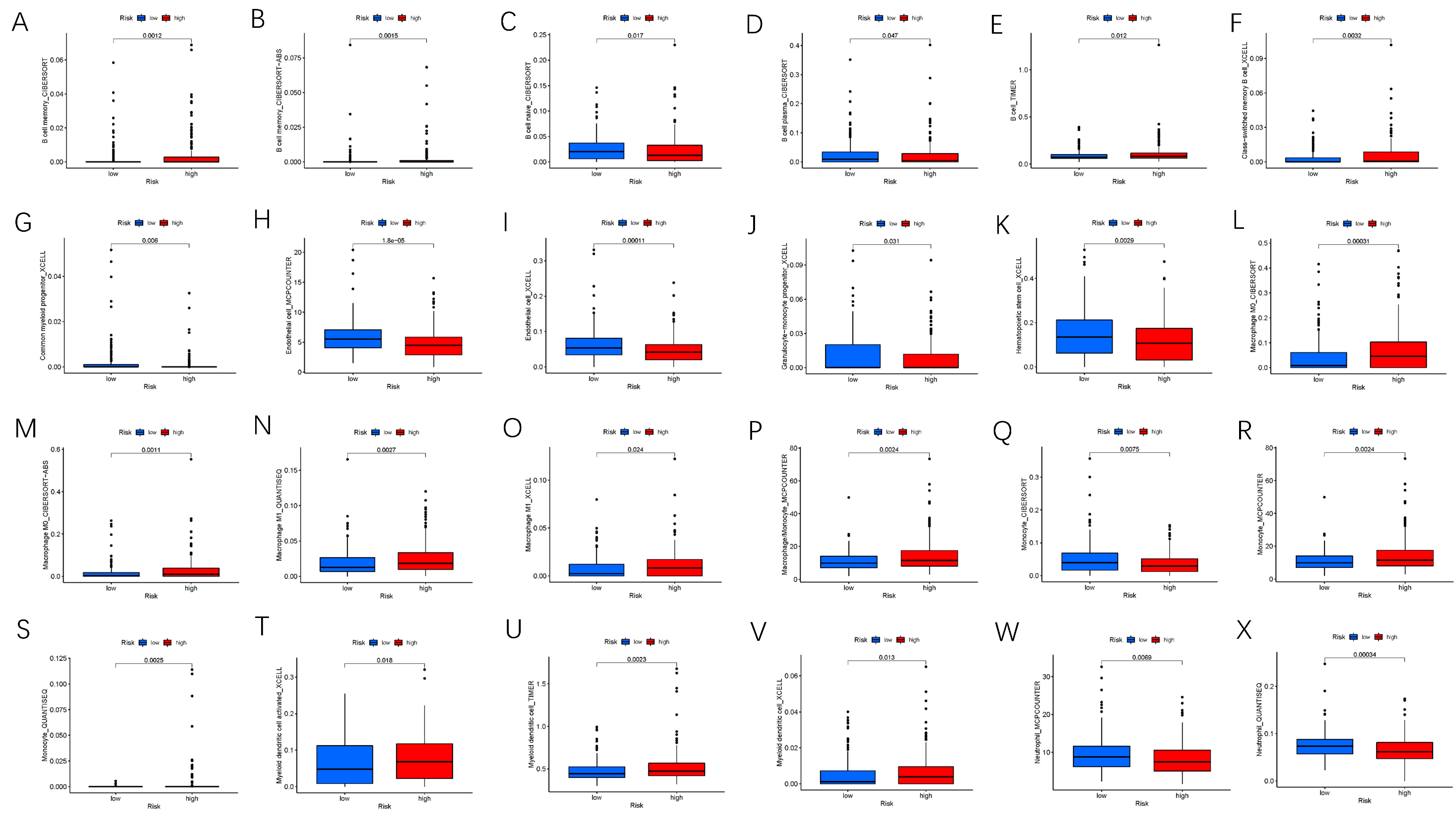

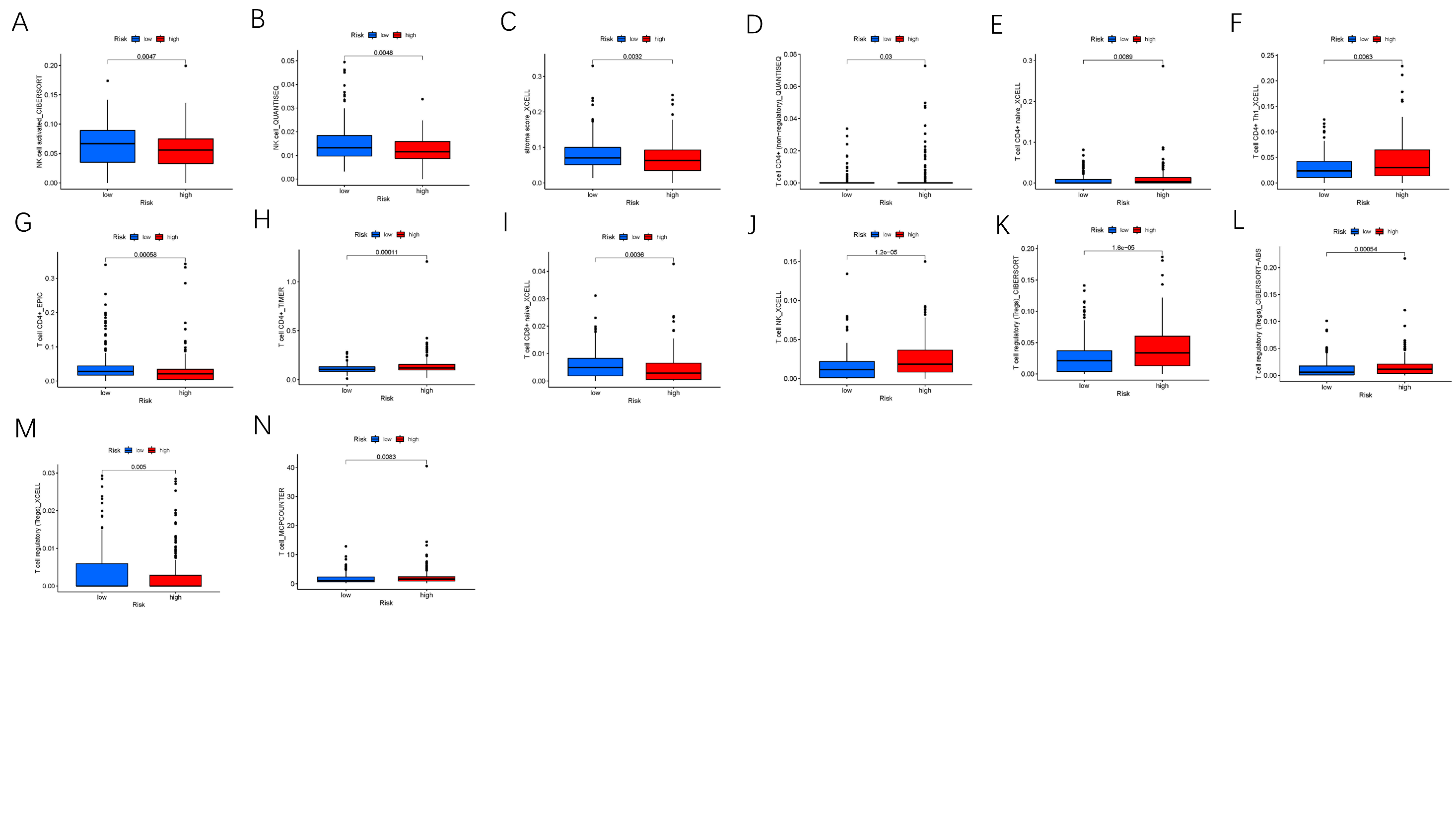
Figure S9: Correlation of prognostic risk score with mutation of genes**. (A) The proportion of mutation of TTN between two risk score subgroups. (B) Kaplan-Meier curves for patients stratified by both mutation of TTN and risk score.


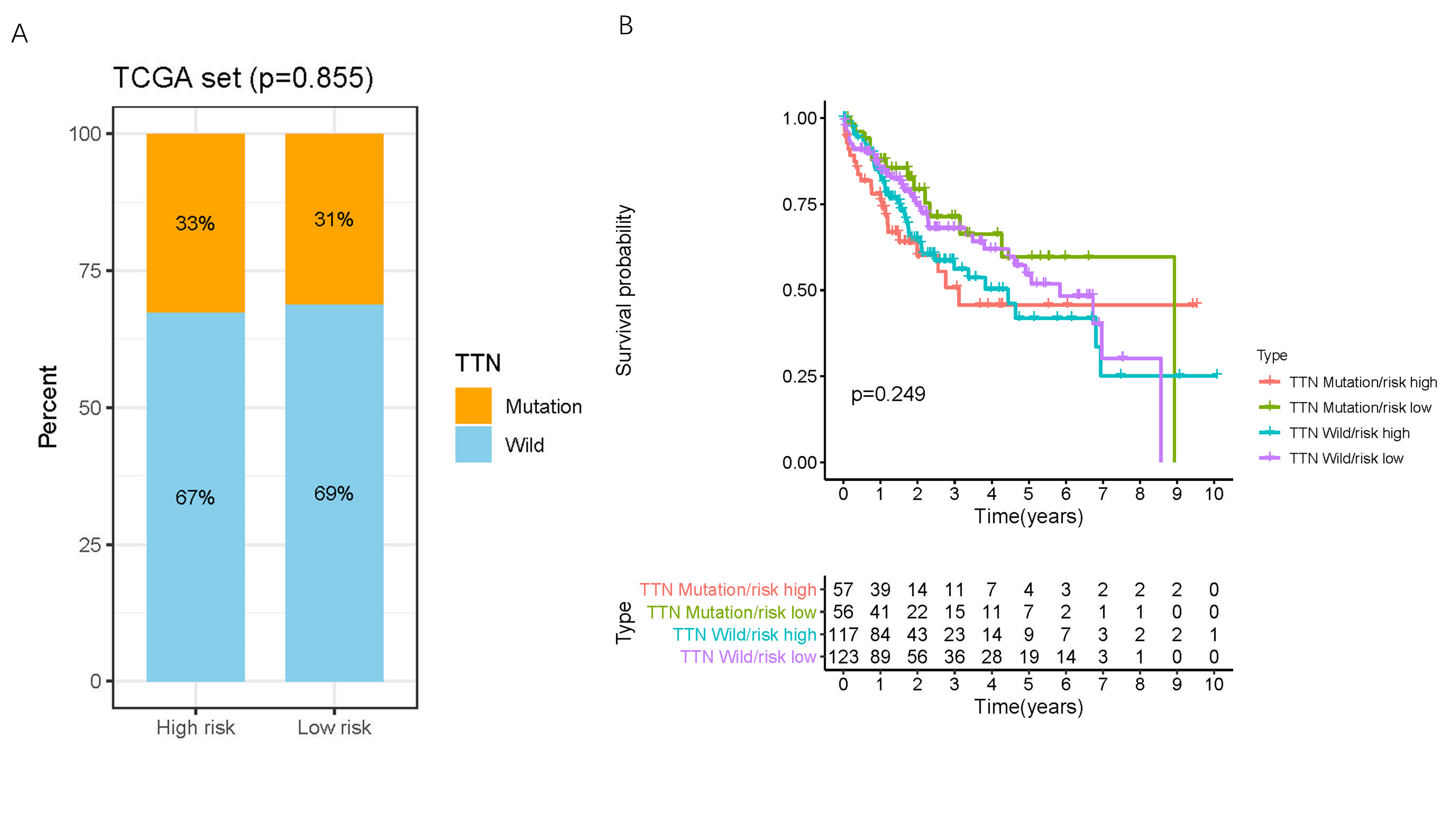

Supplement: Supplementary file 2 — Additional file 2. Supplementary Figure S1. Overall research design. Flow-process diagram presenting the process of comprehensive analysis. Supplementary Figure S2: Prognostic analysis of TMB and correlation with clinical characteristics. (A) Higher TMB levels correlated with better survival outcomes though P>0.05. (B–E) No significant difference of TMB levels was observed with clinical grade, AJCC stage, T status and M status. Supplementary Figure S3: Regression coefficient diagram based on LASSO algorithm. (A) LASSO coefficient profiles of 75 hub genes. A vertical line is drawn at the value chosen by 10‐fold cross‐validation. (B) Ten‐time cross‐validation for tuning parameter selection in the lasso regression. The vertical lines are plotted based on the optimal data according to the minimum criteria and 1-standard error criterion. The left vertical line represents the 3 hub genes finally identified. Supplementary Figure S4: Confirmation of risk score in the external validation group. (A) Heatmap presents the expression pattern of three hub genes in each patient. (B) Distribution of multi-genes signature risk score. (C) The survival status and interval of HCC patients. (D) Kaplan–Meier curve analysis presenting difference of overall survival between the high-risk and lowrisk groups. (E) ROC analysis was employed to estimate the prediction value of the prognostic signature. Supplementary Figure S5: Kaplan–Meier survival analysis for multiple HCC subgroups stratified by clinical variables. (A, B) Age. (C, D) Gender. (E, F) Tumor grade. (G,H) Stage. (I, J) T status. (K) N status. (L) M status. Supplementary Figure S6: (A-C) Areas under curves (AUCs) of the risk scores for predicting 1-, 2-, and 3-year overall survival time with other clinical characteristics. Supplementary Figure S7-S8: The representative results ofthe evaluation of tumor infiltrating immune cells with risk signature. Supplementary Figure S9: Correlation of prognostic risk score with mutation of genes [file 12935_2021_2049_MOESM2_ESM.docx]
